# Integrative Comparison of Variations in Taste, Aroma, and Sensory Characteristics Among Four Sweet Cherry Cultivars to Explore Quality Differences During Storage

**DOI:** 10.3390/foods14193432

**Published:** 2025-10-07

**Authors:** Han Wang, Jingxuan Lu, Luyao Chen, Lizhi Deng, Ranran Xu, Jiankang Cao, Weibo Jiang, Yiqin Zhang, Baogang Wang

**Affiliations:** 1 Institute of Agri-Food Processing and Nutrition, Beijing Academy of Agriculture and Forestry Sciences, Beijing 100097, China; hanwang0201@163.com (H.W.); cly_99@126.com (L.C.); denglizhi0622@163.com (L.D.); xuranran@iapn.org.cn (R.X.); 2 College of Food Science and Nutritional Engineering, China Agricultural University, Beijing 100083, China; lujingxuan1121@126.com (J.L.); cjk@cau.edu.cn (J.C.); jwb@cau.edu.cn (W.J.); 3 College of Food Science, Sichuan Agricultural University, Ya’an 625014, China

**Keywords:** sweet cherry, sensory quality, storage, volatile organic compounds, polyphenolics

## Abstract

The taste, aroma, and sensory characteristics of cherries are key factors influencing consumer acceptance. In this study, the sensory evaluation, biochemical characteristics, and their relationships with consumer satisfaction of several representative cherry cultivars were analyzed during cold storage to establish systematic quality evaluation parameters. Targeted metabolomics analysis revealed significant differences in physiological quality and metabolic profiles among the tested cultivars. Specifically, ‘Benitemari’ demonstrated more contents of soluble solids and titratable acid, while ‘Tieton’ and ‘Skeena’ showed higher concentrations of volatile organic compounds and polyphenolics. Furthermore, hexanal and (E)-2-hexenal were identified as the dominant VOCs, while cyanidin-3-O-rutinoside was confirmed as a major phenolic component across the cultivars. Finally, the comprehensive score of the principal component model was significantly positively correlated with the scores of firmness, chewiness, sweetness, sourness, and taste and bitterness in the sensory evaluation. The results were expected to provide valuable guidance for standardizing the sweet cherry supply chain and cultivating high-quality sweet cherry cultivars.

## 1. Introduction

Cherry fruit (*Prunus avium* L.) is primarily cultivated in temperate zones, appreciated worldwide for its attractive flavor, juiciness, and high nutritional value [1]. Importantly, cherry fruit is beneficial in multiple healthcare functions, such as preventing diabetes, cardiovascular disease, and inflammation, attributed to the bioactive compounds of phenols, vitamins, alkaloids, and others [2,3,4]. However, sweet cherry undergoes vigorous and rapid metabolic activity and senescence after harvest, which leads to a rapid decline in edible quality and nutrient content. Key manifestations of this deterioration include imbalances in sugar, organic acid, and water content, as well as losses of bioactive substances [5].

Currently, cold storage is still mostly used for cherries’ preservation in industry [6]. Unfortunately, inappropriate and long-term low temperature inevitably causes a range of physiological disorders in fruit cells, such as respiration imbalance [7], aroma loss [8], lipid peroxidation [9], and insufficient cell charge. These metabolic disturbances directly contribute to the subsequent deterioration of fruit quality. For instance, the darkening of cherry peel color is mainly caused by enzymatic and non-enzymatic browning [10], the decrease in firmness is primarily attributed to the decomposition of cell wall pectin, a key structural component essential for maintaining tissue integrity [9], and the variation in the ratio of sweetness and sourness is predominantly influenced by alterations in the composition of carbohydrates and organic acids [11]. These factors play a critical role in determining the sensory attributes and overall quality of cherries. Interestingly, the characteristic undesirable bitterness of cherries during later cold storage is associated with plant-derived amino acids, peptides, terpenoids, phenols, flavonoids, and alkaloids [12,13]. Nevertheless, the current evidence on this phenomenon remains insufficient, and further demonstration will benefit from the application of modern mass spectrometry and electronic tongue technologies. Notably, aroma profile has also emerged in recent years as a key factor influencing cherry quality. According to Cozzolino [8], the volatility components of 1-nonanol and 3-pentatone with mint and grass scents disappeared during the later stages of cold storage, which could be related to the metabolism level of flavor precursor substances, including carotenoids, leucine, and octadecenoic acid. The sensory properties of cherry fruit manifest all aspects of color, firmness, sweetness, acidity, and aroma, which have become the main factors in consumer preference and acceptance when choosing products [14].

Currently, it remains a challenge to explore the relationship between consumers’ favorability and fruit quality, which is often ignored in postharvest research. For example, the fruit aroma and peel color occupied a predominant role in the consumer acceptability evaluation. However, in the context of grapes, visual appearance, crispness, and consistency are prioritized as key quality attributes. Similarly, Silva pointed out that sweetness, sourness, and firmness all affect the consumers’ favorability of cherries [12], with interactions among these attributes [15]. Furthermore, López considered that consumer satisfaction was primarily influenced by the fruit’s sugar-acid ratio [14] but also affected by the presence of volatile organic compounds (VOCs), such as ethyl butyrate, (Z)-3-hexene-1-ol, and benzaldehyde. In other words, the texture, color, sugars, acids, phenolics, vitamins, and VOCs constitute the basic quality attributes of cherry fruit, combining to influence the consumers’ favorability and acceptance. In the sensory evaluation conducted by Vavoura [16], the ‘Skeena’ cultivar was reported to achieve the highest flavor score, with high VOCs content, large size, and glossy appearance. Principal component analysis (PCA) concluded that ‘Tieton’ possessed the lowest comprehensive score during storage, indicating its shorter shelf life and poorer quality. Conversely, ‘Nanyo’, classified as a yellow variety, was primarily cultivated in Japan with limited research. ‘Benitemari’ was characterized by its bright red peel color, with few studies on its physiological properties.

To date, recent publications have mainly focused on preservation techniques for cherry fruit, with limited attention paid to the influence of intrinsic fruit quality on sensory perception. Therefore, the present study selected multiple representative cherry cultivars to conduct a comprehensive comparison of their storage characteristics and physiological quality attributes. Special emphasis was placed on illustrating the relationship between these quality parameters and consumer acceptability. This study is expected to provide directions for the cultivation of high-quality cherry varieties from the perspective of consumer acceptance.

## 2. Materials and Methods

### 2.1. Reagents and Fruit Materials

Folin–Ciocalteu reagent, gallic acid, and (+)-catechin were purchased from Sigma-Aldrich Co. (St. Louis, MO, USA). The anthocyanin standard was from Macklin Company (Shanghai, China), and other polyphenolic standards were obtained from Shanghai Yuanye Biological Reagent Co., Ltd. (Shanghai, China). All other chemicals used were of analytical grade.

Sweet cherries (*Prunus avium* L.) in this work included four cultivars, named cvs ‘Benitemari’, ‘Nanyo’, ‘Tieton’, and ‘Skeena’. Their original resources and basic quality attributes were shown in Table S1. The cherry fruit was harvested at commercial maturity stage (the peel color of black-cherry cultivars (‘Tieton’ and ‘Skeena’) received blackish red and uniform, light-colored cultivars (‘Benitemari’ and ‘Nanyo’) was evenly covered with red and yellow; and the stems were bright green and plump) in July 2023 and transported carefully to the laboratory at China Agriculture University within 2~5 d to avoid mechanical damage. Before treatment, sweet cherries with signs of decay and physical damage were discarded. Sweet cherries were randomly selected and placed in perforated polyethylene plastic boxes (160 × 120 × 60 mm), with 30 cherries per box. For each cultivar, the cherries were randomly divided into 3 independent batches, where each batch consisted of 8 boxes. The first batch was immediately analyzed to determine the initial fruit characteristics at harvest, and the other two batches were stored at 0 °C, 95% RH and analyzed at 10 and 20 days. For metabolome extraction, twenty fruits per batch with pits removed were immediately frozen with liquid nitrogen, powdered, and maintained at −80 °C.

### 2.2. Evaluation of Sensory Quality

The sensory analysis of cherry fruit was evaluated by a twenty-member trained panel according to the method described by López with modifications [14]. Before consumer sensory evaluation, cherries were removed from cold storage and allowed to equilibrate at room temperature until the fruit pulp reached 25 °C (with no condensation water on the peel). For each cherry cultivar, samples were labelled with a random three-digit reference code to avoid panel bias. Each sample consisted of five intact fruits, which were served in white polystyrene plates, and the order of sample presentation to panelists was randomized to eliminate sequence effects. The panel was instructed to score the sensory descriptors that determined the fruit size, color, fruit shape, firmness, chewiness, sweetness, sourness, juiciness, taste and bitterness, and overall satisfaction. In order to standardize the panel, two commercially common cherry cultivars (cvs. ‘Summit’ and ‘Brooks’, sourced from a local market) with well-documented sensory traits were used as reference standards. For overall acceptability, the panel was asked to indicate their degree of liking/disliking by a nine-point hedonic scale (1 for extreme dislike and 9 for extreme like) for their satisfaction. All sensory evaluations were performed at room temperature in individual testing booths under uniform white fluorescent lighting.

### 2.3. Determination of Basic Quality Characteristics

The quality assessment of cherry fruit was conducted according to our previous methods with some modifications [9,17,18]. A total of 30 cherries were selected to measure soluble solids content (SSC) with a refractometer (ATAGO, Tokyo, Japan), and the measured values were expressed as %. For the determination of soluble sugar content, 1.0 g of frozen sample was analyzed using the sulfuric acid-anthrone method, and the result was expressed as g kg^−1^ of sucrose. Titratable acid (TA) was determined by titrating 3.0 g of fruit homogenate with 0.01 mol L^−1^ NaOH (end point of pH 7.5) via an automatic acid-base titrator (INESA, Shanghai, China), and the result was expressed as the percentage of malic acid. For fruit firmness determination, 30 cherries were tested to measure penetration force on the fruit flesh using a 6 mm-probe penetrometer (FT-02; Fruit Test Co., Ltd., San Giovanni, Italy), and the data were expressed in N. The peel color of 60 cherries was determined using a reflectance spectrophotometer to reduce individual differences (Model NF333, Nippon Denshoku Industries, Tokyo, Japan), and the result was expressed according to the CIE Lab system (a* as red/green, b* as yellow/blue). All the results were expressed on a fresh weight basis.

### 2.4. Analysis of Soluble Sugars and Organic Acids

The extraction and determination of sugars and organic acids were performed according to the previous conditions with some modifications [19]. For sugars, 1.0 g of frozen sample with 10 mL 95% (*v*/*v*) ethanol was ultrasonically extracted at 30 °C for 1 h and centrifuged at 12,000 rpm for 20 min. All the supernatants were passed through a 0.22 μm membrane filter (Millipore, Bedford, MA, USA) before high-performance liquid chromatography (HPLC) analysis (Waters, Milford, OH, USA). For the analysis of sugars, an Agilent Carbohydrate Chromatography column (5 μm, 4.6 mm × 250 mm) was employed, the mobile phase was set as acetonitrile-water (75:25, *v*/*v*), and the chromatographic conditions were configured as follows: a flow rate of 1.0 mL min^−1^, a column temperature of 30 °C, and a sample injection volume of 10 μL. For organic acids, 1.0 g frozen sample was ground in 20 mL deionized water and shaken at 25 °C for 1 h, and then centrifuged at 12,000 rpm for 20 min. For organic acids, the analytical column used was an ACE C18 column (5 μm, 4.6 mm × 250 mm), with the mobile phase consisting of 1000 mL of 0.01 M phosphate buffer (pH = 2.3) and 20 mL of methanol, and the chromatographic conditions were set as follows: a flow rate of 1.0 mL·min^−1^, a column temperature of 35 °C, a sample injection volume of 10 μL, and a detection wavelength of 214 nm.

Sugars and organic acids were qualitatively and quantitatively determined by comparing the retention time and peak area of standard substances, including glucose, fructose, sucrose, maltose, lactose, malic acid, succinic acid, citric acid, tartaric acid, and fumaric acid. For the quantitative determination of soluble sugars and organic acids, individual calibration curves were constructed for each target analyte to ensure analytical accuracy. With respect to sugars, glucose and fructose were calibrated using serial concentration gradients of 20, 40, 60, 80, and 120 g/kg; sucrose and maltose with gradients of 10, 30, 50, 70, and 90 × 10^−2^ g/kg; and lactose with gradients of 6, 12, 18, 24, and 30 × 10^−3^ g kg^−1^. For organic acids, malic acid was calibrated using gradients of 5, 10, 15, 20, and 25 mg/kg; succinic acid and citric acid with gradients of 0.4, 0.8, 1.2, 1.6, 2.0, and 2.4 mg kg^−1^; tartaric acid with gradients of 0.5, 1.0, 1.5, 2.0, and 2.5 × 10^−1^ mg/kg; and fumaric acid with gradients of 2, 4, 6, 8, and 10 × 10^−3^ mg/kg. All standard solutions were prepared using extraction-matched solvents to minimize matrix effects, and each concentration level was analyzed in triplicate to validate the reliability and reproducibility of the calibration curves. The results were expressed as g kg^−1^.

### 2.5. Analysis of Volatile Organic Compounds

Then VOCs in samples were detected via gas chromatography–mass spectrometry/mass spectrometry (GC–MS/MS-2030, Shimadzu, Kyoto, Japan) [17]. Specifically, 3.0 g tissue samples, 1.0 mL saturated NaCl, 3.0 g cross-linked Polyvinylpolypyrrolidone (PVPP), and 10 μL 2-octanol (100 mg kg^−1^, internal standard) were added to a 20 mL airtight headspace vial. Then, a 50/30 μm divinylbenzene/carboxen/polydimethylsiloxane (DVB/CAR/PDMS) fiber was inserted into the airtight headspace vial, and the vial was maintained at 45 °C with a stirring speed of 500 rpm for 30 min for adsorption. Subsequently, the fiber was placed into a GC-MS analysis program for desorption and analysis. The GC analysis conditions were set as follows: column (DB-5 ms, 30 m × 0.25 mm × 0.25 μm) was used with a programmed column temperature (initial temperature of 40 °C for 2 min, then increased to 200 °C at a rate of 8 °C·min^−1^, and finally increased to 250 °C at a rate of 10 °C·min^−1^ (held for 2 min); and the total analysis duration was 59 min, and high-purity helium (He) was used as the carrier gas at a flow rate of 1.0 mL·min^−1^. The front inlet was configured in splitless mode, with a purge flow rate of 6.0 mL·min^−1^ and an injection temperature maintained at 250 °C. The MS conditions were as follows: ion source temperature of 230 °C, quadruple temperature of 150 °C, electron ionization (EI) energy of 70 eV, and a mass scanning range of 30–500 *m*/*z*. Each sample was repeated in triplicate to ensure reproducibility.

VOCs were preliminarily identified by matching degree, retention time, and retention index (RI) according to the NIST 08 database (National Institute of Standards and Technology), with additional validation using data from related publications. For quantification, the peak areas of extracted ions corresponding to each VOC were used, and a semi-quantitative approach was adopted to calculate the relative content of individual VOCs in cherry samples using the peak area of an internal standard as the reference benchmark. The results were expressed as mg kg^−1^.

### 2.6. Determination of Total Phenolics, Total Flavonoid Contents, and Antioxidant Capacity

The determination of total phenolic contents (TPC) and total flavonoid contents (TFC) was performed according to previously Folin–Ciocalteu and aluminum salt chromogenic methods [9,20]. The results were expressed as mg kg^−1^ with gallic acid for TPC and catechin for TFC as the standard substances, respectively.

Antioxidant capacity is characterized mainly by free radical scavenging ability, we used the 2,2-diphenyl-1-picryhydrazyl (DPPH) free radical scavenging ability assay kit (Beijing Boxbio Science & Technology Co., Ltd., Beijing, China) and 2,2′-amino-di (2-ethyl-benzothiazoline sulphonic acid-6) ammonium salt (ABTS) free radical scavenging ability assay kit (Beijing Boxbio Science & Technology Co., Ltd., China), the result was expressed as %.

### 2.7. Analysis of Phenolic Compounds

The extraction and determination of phenol compounds were performed referring to the study of Panić with slight modifications [21], via the HPLC system equipped with a diode array detector (DAD) and a Phenomenex C18 column (Kinetex (Bangkok, Thailand), 2.6 µm × 150 mm × 4.6 mm). HPLC analysis was performed using a mobile phase composed of solvent A (water/formic acid, 99.9:0.1, *v*/*v*) and solvent B (methanol/formic acid, 99.9:0.1, *v*/*v*), with a gradient elution program for solvent B set as follows: maintaining 10% solvent B from 0 to 3 min, linearly increasing solvent B from 10% to 50% over the period of 3 to 15 min, further elevating it from 50% to 60% between 15 and 20 min, continuing to raise it from 60% to 100% during 20 to 25 min, and holding at 100% solvent B for 1 min (25 to 26 min); the flow rate of the mobile phase was fixed at 1.0 mL min^−1^, the column temperature was controlled at 30 °C to ensure stable chromatographic separation, and the autosampler was maintained at 4 °C to preserve the stability of the injected samples before analysis. Phenolic compounds were qualitatively and quantitatively determined by comparing the retention time and peak area of the standard substances, including caffeic acid, chlorogenic acid, neochlorogenic acid, ferulic acid, p-coumaric acid, quinic acid, vanillic acid, catechin, epicatechin, rutin, kaempferol, and naringenin. The results were expressed as g kg^−1^.

### 2.8. Analysis of Anthocyanin Components

Referred to the description by Dong with slight modifications [22], the HPLC (Agilent, San Jose, CA, USA) was used to determine the anthocyanin components of the cherries. Briefly, anthocyanins were extracted from 0.5 g of ground frozen fruit samples by the ultrasonic extraction method (10 min, 120 W, 40 °C) in dark conditions using 10 mL of 3% (*v*/*v*) formic acid-methanol. Then the mixture was centrifuged at 12,000 rpm for 15 min. Repeated the extraction process twice and combined the supernatant. The total extraction was then filtered through a 0.22 μm PTFE (polytetrafluoroethylene) filter for subsequent HPLC analysis (equipped with a VWD detector and SB-Zobax C18 column, 5 μm × 250 mm × 4.6 mm). HPLC analysis was conducted using a mobile phase composed of acetonitrile and 5.0% formic acid solution at a fixed volume ratio of 24:76, with a constant flow rate of 1.0 mL min^−1^, an injection volume of 10 μL, a column temperature maintained at 25 °C to ensure stable chromatographic behavior, and a detection wavelength set at 520 nm for selective quantification of target analytes.

### 2.9. Statistical Analysis

The results were performed in triplicate for each experiment and expressed as mean ± standard deviation. The statistical analysis was conducted by using IBM SPSS Statistics software Version 19 (SPSS Inc., Chicago, IL, USA) at 5% level of significance. One-way analysis of variance (ANOVA) and Duncan’s test were used to assess the data.

## 3. Results and Discussion

### 3.1. Analysis of Basic Quality for Different Cherry Cultivars

#### 3.1.1. SSC and Soluble Sugar

**Figure 1:**
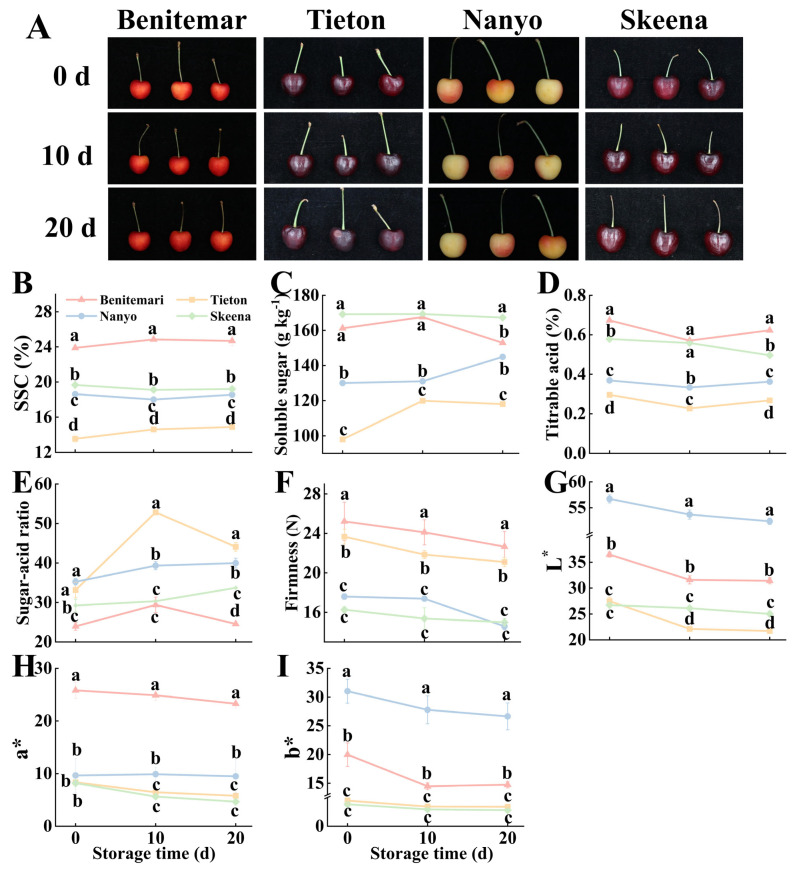
Changes in appearance of cherry fruits (**A**), Soluble solid contents (**B**), Soluble sugar (**C**), Titratable acid (**D**), Sugar−acid ratio (**E**), Firmness (**F**), L* (**G**), a* (**H**), b* (**I**) of four different cultivars of cherry during storage at 0 °C for 20 d. Vertical bars represent the standard error of the means. Different letters indicate significant differences (ANOVA, *p* < 0.05) between four different cultivars of cherry on the same day.

SSC and soluble sugar content represent the sweetness of cherries, directly affecting consumer satisfaction [14]. The appearance of each cherry cultivar was clearly presented in Figure 1A. From Figure 1B,C, the levels of soluble solids and soluble sugar in ‘Skeena’ were particularly high (19.67% and 169.17 g kg^−1^, respectively), significantly (*p* < 0.05) surpassing those in ‘Tieton’ (13.55% and 98.03 g kg^−1^, respectively), consistent with the results in Gonçalves [23]. Notably, while the ‘Benitemari’ cherry cultivar exhibited the highest SSC (soluble solids content), its soluble sugar content did not rank the highest among all tested cultivars. The variation trends of SSC and soluble sugar content differed among cherry cultivars, and inter-cultivar difference is primarily related to the intrinsic cultivars’ characteristics and postharvest metabolic activities, such as cellular water loss, starch hydrolysis, cell wall polysaccharide hydrolysis, respiration, and transpiration [22,24]. These directly or indirectly regulated the accumulation and redistribution of soluble carbohydrates, thereby shaping the final SSC and soluble sugar content profiles of different cultivars.

#### 3.1.2. TA

TA affects the acidity of cherries, which is one of the most important reference indicators for judging the cherries’ taste [14]. From Figure 1D, the content of TA ranged from 0.23% to 0.67%. Furtherly, the lowest TA value was found in ‘Tieton’ (0.23%), which was lower than the range measured by Vavoura [16]. The decrease in TA may be linked to the altered respiratory metabolic changes in organic acids inside the fruit, while the increase might stem from cherry moisture loss—reduced water content relatively elevates organic acid concentration, leading to higher measurable TA, consistent with previous findings [25].

#### 3.1.3. Sugar-Acid Ratio

The sugar-acid ratio is recognized as one of the key comprehensive indices for evaluating the maturity and edible quality of sweet cherries [26]. As shown in Figure 1E, the sugar-acid ratio of cherry was distributed in the range of 23.95–52.84, with statistically significant differences (*p* < 0.05) observed among the four cultivars. Among all analyzed samples, the highest ratio was found in ‘Tieton’, but the SSC was the lowest, indicating the relationship between sugar-acid ratio and SSC was weak, and no consistent trend in the sugar−acid ratio and SSC relationship was observed across the tested cultivars. These findings are consistent with those previously reported by Chockchaisawasdee [27].

#### 3.1.4. Firmness

Fruit firmness is closely associated with consumer satisfaction, which is widely used as a key indicator to evaluate the quality and guide selective harvesting [27]. As shown in Figure 1F, the firmness of the tested cherries exhibited statistically significant differences (*p* < 0.05), ranging from 14.58 N (cultivar ‘Nanyo’ at 20 d) to 25.21 N (cultivar ‘Benitemari’ at 0 d). Interestingly, ‘Skeena’ performed the lowest firmness value, while ‘Nanyo’ performed the highest value, a variation primarily attributed to inherent genetic differences among cultivars [28]. During storage, a consistent decline in firmness was observed across all cultivars, with ‘Tieton’ exhibiting the most rapid rate of firmness loss. The decrease in the firmness value was widely attributed to the degradation of pectin in the fruit cell wall [9]. Specifically, at the later stages of storage, the activity of pectinase increased significantly; this elevated enzyme activity accelerates pectin decomposition, which in turn weakens intercellular adhesion and reduces the mechanical strength of the cell wall. Ultimately, these physiological changes lead to a marked decrease in sweet cherry firmness [4].

#### 3.1.5. Color Attributes

Fruit color is a critical indicator for assessing sweet cherry maturity and freshness [15], as it is one of the primary criteria guiding consumer purchase decisions. L*, a*, and b* values represent brightness, red, and yellow in CIE systems, respectively. As shown in Figure 1I, the analysis of color parameters indicated that ‘Tieton’ exhibited the darkest fruit, as indicated by its lowest L* value (21.47), while ‘Nanyo’ displayed a yellow appearance, reflected in its highest L* value (higher L* index, 56.71). Most of the L* values obtained in this study were consistent with previous reports [23], which documented L* values between 18.95 and 43.30. However, the L* value of ‘Nanyo’ (56.71) was substantially higher than the range reported in that work.

Among the tested cultivars, the lowest a* and b* values were found in ‘Skeena’ (4.66, 2.80, respectively), which corresponded to the deepest/darkest fruit coloration. In contrast, ‘Benitemari’ exhibited the highest a* value (25.81), indicating the most intense red coloration among all examined cultivars. ‘Nanyo’ cherries showed the highest b* value (31.02), and statistical analysis confirmed that ‘Nanyo’ had statistically significant (*p* < 0.05) color differences compared to the other cultivars. During storage, a consistent decrease in L*, a*, and b* values was observed across all cultivars—a trend consistent with findings from other studies [13]. This reduction in color parameter values was thought to be associated with the catabolism of anthocyanins, as well as changes in the pH and ion concentration of the fruit tissue.

#### 3.1.6. Total Phenolics Content, Total Flavonoids Content, and Antioxidant Capacity

**Figure 2:**
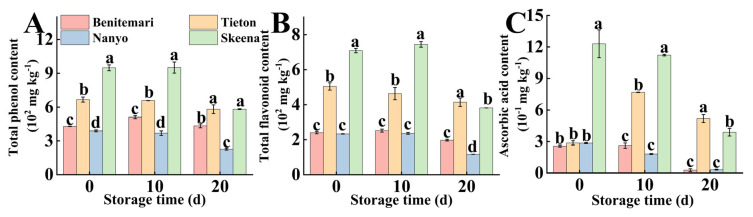
Changes in Total phenolic contents (**A**), Total flavonoids contents (**B**), and Ascorbic acid content (**C**) of four different cultivars of cherry during storage at 0 °C for 20 days. Vertical bars represent the standard error of the means. Different letters indicate significant differences (ANOVA, *p* < 0.05) between four different cultivars of cherry on the same day.

As important secondary metabolites in plants, total phenolics and flavonoids play a crucial role in regulating fruit physiological processes. They also exhibit prominent antioxidant and anti-inflammatory activities, which are well-documented to confer health benefits to humans. Notably, the contribution of phenolics and flavonoids has been extensively investigated in various fruit species, including persimmons, bananas, and grapes [2]. This underscores their significance in fruit sensory quality, prompting the recognition of total phenolics and flavonoid concentrations as key indicators of flavor quality in cherries. In the present study, total phenolic contents (TPC) and total flavonoid contents (TFC) of different cherry cultivars, as illustrated in Figure 2A,B, followed similar variation trends. This observation aligns with previous findings by Di Matteo [29], who reported consistent patterns in phenolic and flavonoid concentrations across different fruit varieties.

Ascorbic acid also exhibited prominent antioxidant activity, which enabled the scavenging of free radicals accumulated in plant cells, further retarded the progression of fruit oxidation, thereby contributing to the preservation of both chromatic stability and characteristic flavor profiles of fruits. Similarly, as shown in Figure 2C, the ascorbic acid content of ‘Skeena’ was significantly higher than that of the other cultivars. However, the ascorbic acid content decreased rapidly in the late stage of storage, a trend that was consistent with the changes in total phenol and total flavonoid contents. During postharvest storage, ascorbic acid is prone to degradation, with this process being primarily driven by temperature fluctuations, light exposure, and the activity of endogenous enzymes [7]. Notably, for the ‘Tieton’ cultivar, an increase in ascorbic acid content was observed on the 10th day of storage, a phenomenon hypothesized to be associated with the dynamic balance between the activities of synthesizing enzymes and degrading enzymes of ascorbic acid. In the early postharvest stage, the rate of ascorbic acid synthesis temporarily exceeds its degradation rate in ‘Tieton’, ultimately leading to a stage-specific accumulation of ascorbic acid during this storage period.

Significantly, the free radical scavenging capacity of DPPH and ABTS (Table 1) demonstrated the same variation trend as those of TPC and TFC, indicating that the antioxidant capacity was primarily dependent on their TPC and TFC levels [29]. Among all the tested cultivars, ‘Skeena’ exhibited the highest TPC and TFC with values reaching 951.28 mg kg^−1^ and 744.53 mg kg^−1^, respectively, at 10 d. These values were significantly higher than those reported for other sweet cherry cultivars in previous studies [30]. In contrast, the lowest values were observed in ‘Nanyo’ (226.15 mg kg^−1^, 116.43 mg kg^−1^, 20 d), which were comparable to those reported for less astringent varieties [13]. Collectively, these results confirm that TPC and TFC in sweet cherries are influenced by multiple factors, including cultivar genotype, climatic conditions, cultivation practices, and postharvest storage conditions [28].

As illustrated in the figure, the contents of total phenolic compounds (TPC), total flavonoids (TFC), and ascorbic acid in the dark-colored sweet cherry cultivars ‘Skeena’ and ‘Tieton’ were significantly higher than those in the light-colored cultivars (*p* < 0.05). This observation is closely associated with the anthocyanin composition that determines sweet cherry peel color. The peel color of sweet cherries was primarily regulated by phenolic compounds, among which anthocyanins (e.g., cyanidin-3-O-rutinoside, a major anthocyanin component in sweet cherries) are the key contributors to the dark color of the peel [10]. Consistent with this, the dark-colored cultivars (‘Skeena’ and ‘Tieton’) exhibited a notably higher anthocyanin content compared to their light-colored counterparts (Table 2).

Notably, phenolic compounds (including flavonoids) and ascorbic acid are well-recognized as primary contributors to the antioxidant capacity of fruits [15]. Given the higher levels of TPC, TFC, and ascorbic acid in dark-colored sweet cherries, it follows that these cultivars also displayed significantly stronger antioxidant capacity than the light-colored ones. This finding further confirmed that the darker peel color of cherries was not only a visual indicator of higher anthocyanin accumulation but also a potential marker for antioxidant-related nutritional quality.

### 3.2. Analysis of Soluble Sugar and Organic Acid Components of Different Cherry Cultivars

Previous studies have reported that sweet cherry fruit contains a range of primary metabolites, such as carbohydrates, and especially simple sugars (sucrose, glucose, fructose), as well as organic acids (citric acid and malic acid) [31]. Sweetness and acidity are widely recognized as core indicators for assessing the quality and taste of fresh fruits. Specifically, research has demonstrated that the sugar composition of sweet cherries remains relatively stable during postharvest handling or storage, and fructose has been identified as the primary contributor to the fruit’s sweetness [32]. For organic acids, even small or trace amounts play important roles; they not only act as effective additives to enhance food flavor but also serve as potential precursors for the biosynthesis of other flavor-related compounds in sweet cherries [31].

**Figure 3:**
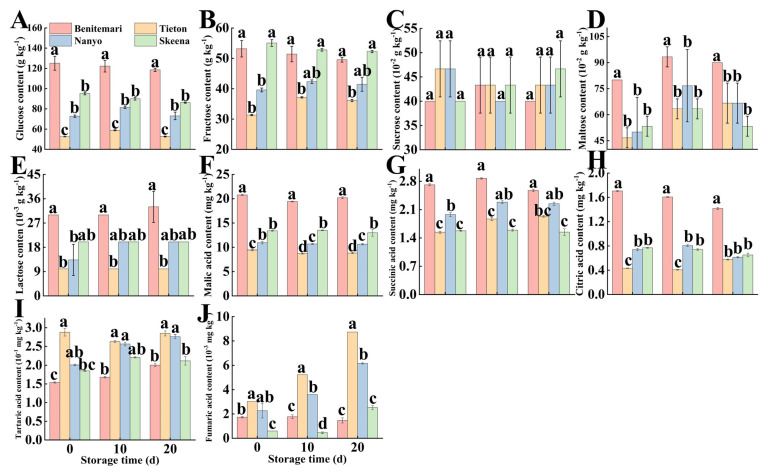
Changes in glucose content (**A**), Fructose content (**B**), Sucrose content (**C**), Maltose content (**D**), Lactose content (**E**), Malic acid content (**F**), Succinic acid content (**G**), Citric acid content (**H**), Tartaric acid content (**I**), Fumaric acid content (**J**) of four different cultivars of cherry during storage at 0 °C for 20 d. Vertical bars represent the standard error of the means. Different letters indicate significant differences (ANOVA, *p* < 0.05) between four different cultivars of cherry on the same day.

In the analyzed cultivars (Figure 3), five soluble sugars were identified: glucose, fructose, sucrose, maltose, and lactose. Among these, glucose and fructose were the predominant soluble sugar components (Figure 3A,B), with their concentrations being statistically significantly higher (*p* < 0.05) than those of the other three sugars, aligning with previous findings [33]. Obviously, the highest level of glucose (55.07 g kg^−1^) was contained by ‘Skeena’, while the highest concentration of fructose (125.10 g kg^−1^) was exhibited in ‘Benitemari’. From Table S2, ‘Benitemari’ exhibited the highest soluble sugar content, whereas ‘Tieton’ had the lowest soluble sugar content, indicating significant cultivar-specific differences in the accumulation of soluble sugars. Furthermore, glucose and fructose were also lower in ‘Tieton’. In addition, fructose primarily influenced the sweetness of sweet cherries, as documented in prior literature [34].

Organic acids are critical for the development of fruit flavor and directly determine the overall flavor quality of sweet cherries [5]. A total of six organic acids (malic acid, succinic acid, citric acid, tartaric acid, ascorbic acid, and fumaric acid) were detected by HPLC analysis (Figure 3). Among these organic acids, malic acid was the most abundant component across all cultivars, a trend particularly prominent in ‘Benitemari’ and ‘Skeena’ (Figure 3F). This observation confirms the findings of previous studies, which also identified malic acid as the dominant organic acid in sweet cherries [31]. In contrast, tartaric acid was least accumulated, only up to 0.29 g kg^−1^ (Table S2), and this level was substantially lower than the maximum concentration of malic acid (20.79 g kg^−1^). These results collectively indicate that the acidity of sweet cherries is primarily determined by malic acid.

### 3.3. Analysis of Volatile Organic Compounds Profiles of Different Cherry Cultivars

**Figure 4:**
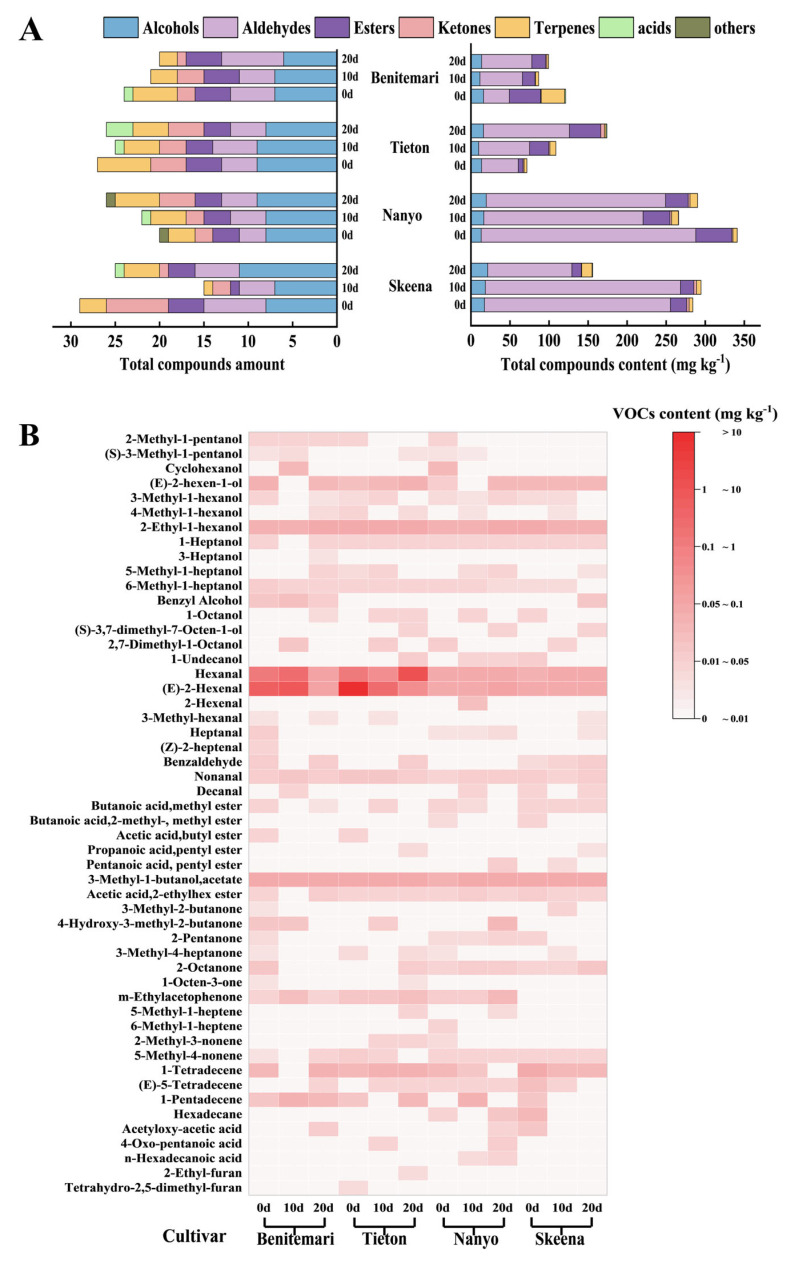
Changes in total volatile compounds amount and total volatile compounds concentration (**A**) and Heat map volatiles concentration (**B**) of four different cultivars of cherry during storage at 0 °C for 20 d.

It is well established that cultivar genotype exerts a significant influence on the metabolic composition of sweet cherries, which in turn directly affects the fruit’s nutritional value and sensory quality [33]. However, now only a few cultivars have been comprehensively characterized for these important features. In order to establish the metabolic fingerprint for the evaluated cherry cultivars, a comparative analysis of the major metabolites was conducted. Specifically, a total of 52 VOCs were identified and relatively quantified by SPME GC−MS in the sweet cherry samples. From Figure 4A, the metabolites can be divided into six different chemical categories: esters (7), alcohols (16), aldehydes (9), ketones (7), terpenes (8), acids (3), and others (2). Among all the tested cultivars, ‘Benitemari’ exhibited the highest VOC diversity, with 41 compounds identified.

As presented in Table S3, the aroma description of the volatile compound was also demonstrated. The relative content of each compound was determined based on the IS (inter standard) peak area. From Figure 4B, the dominant VOCs across the different sweet cherry cultivars were E-2-hexenal and hexanal. This observation is consistent with the findings of previous studies on sweet cherry aroma profiles [30]. In addition to these two primary VOCs, other key volatile components, including 2-ethyl-1-hexanol, E-2-hexen-1-ol, and 3-methylbutyl acetate, were also the main aroma components affecting the flavor of sweet cherries.

From Table S3, a total of 16 alcohols were identified, which mainly conferred cherries with floral and fruity aroma. Among them, cyclohexanol, (E)-2-hexen-1-ol, 2-ethyl-1-hexanol, and benzyl alcohol were detected at relatively high levels. And these compounds were related to the metabolic transformation of the high-concentration aldehydes mentioned, such as hexanal and E-2-hexenal [30], and the latter, respectively, impart camphoraceous, fruity, herbaceous, sweet, and floral aromas to cherries. Notably, E-2-hexen-1-ol was detected in all four cultivars and exhibited increased levels during storage. And the same goes for 2-ethyl-1-hexanol, with the highest relative concentration of up to 15 mg kg^−1^ (‘Benitemari’, 20 d), significantly higher than the other aroma components. In contrast, cyclohexanol was present only in ‘Benitemari’ and ‘Skeena’, both of which disappeared at 20 d, and it may be converted cyclohexanol was only present in ‘Benitemari’ and ‘Skeena’ and was not detected in either cultivar at 20 d of storage; this disappearance may be due to its conversion into other aroma components such as C6-OH. Similarly, benzyl alcohol was also limited to ‘Benitemari’ and ‘Skeena’, and the presence of 1-undecanol added a distinct rose aroma to the cherries. Alcohols, which are by-products of unsaturated fatty acid oxidation, are synthesized via the alcohol dehydrogenase-mediated reduction of aldehydes. Specifically, linolenic acid is oxidized by lipoxygenase-catalyzed metabolism to produce 2 E or 3 (E or Z) hexenal, which is reduced to 2 E or 3 (E or Z) hexanol [35].

Notably, aldehydes were the predominant VOCs across all cherry cultivars, accounting for more than 50% of the total content, contributing to the main aroma of sweet cherries, which was primarily associated with green-grassy and orange. A total of 9 aldehydes were identified, with hexanal and E-2-hexenal being the most abundant. Specifically, the relative concentration of hexanal reached 114.84 mg·kg^−1^ in ‘Benitemari’ at 10 d of storage, while that of (E)-2-hexenal peaked at 169.82 mg·kg^−1^ in ‘Tieton’ at 0 d (initial storage). Both compounds were detected in all four cultivars and served as core aroma constituents, imparting a distinct green-grassy fragrance to the fruits. Nonanal was also present in different cultivars of cherries, ranging from 0.27 to 1.33 mg kg^−1^, with higher levels in ‘Tieton’, which contributed a grassy and fresh aroma, resulting from the decomposition of oleic acid by hydrogen peroxide [8]. Benzaldehyde, which was present in almost cultivars, was also a major contributor to the characteristic flavor of the cherries, providing a pleasant aroma reminiscent of sweet almonds, burnt sugar, and caramel. These aldehydes were widely available, and some of them have been previously identified in berries, including strawberry, blueberry, and pomegranate [35]. In terms of biosynthesis, VOCs associated with green flavors are primarily linked to C6 aldehydes and alcohols—especially their unsaturated derivatives. Specifically, C6 and C9 aldehydes are mainly produced through the α- and β-oxidation-mediated degradation of fatty acids; among these, hexenal is synthesized from linolenic acid via the lipoxygenase metabolic pathway [36].

Esters, as essential aroma compounds, contributed to the wine and fruit odor of cherries. Notably, significant differences in the content of ester compounds were observed among the different cherry cultivars evaluated in this study. 3-Methyl-1-butanol acetate was identified as the predominant ester component, a finding consistent with previous reports [37]. And it was found in four cherry cultivars with a relative content of 5.57–45.58 mg kg^−1^, which endowed cherries with the aroma of wine, fruit, and grass. Similarly, acetic acid, 2-ethylhexyl ester, was present in every cultivar; its presence may be related to the accumulation of 2-ethyl-1-hexanol, with a fruity and pear aroma. Among the tested cultivars, most types of ester compounds were contained in ‘Skeena’, while the highest content of esters was found in ‘Tieton’. Interestingly, the total esters relative contents of ‘Nanyo’ increased during storage, which may be associated with the senescence process of fruit [8]. In addition, accumulating evidence indicates that C5, C6, and C9 volatile compounds in horticultural crops (e.g., vegetables and fruits) are synthesized via the lipoxygenase (LOX) pathway, which involves the β-oxidation of fatty acid precursors [35,38].

For terpenes, advanced olefins emerged as the primary aromatic components of eight terpenes. Specifically, 1-tetradecene (0–24.03 mg kg^−1^) and 1-pentadecene (0–5.76 mg kg^−1^) were the main aroma components with high levels, aligning with other studies [37]. Notably, 5-methyl-1-heptene (0–0.43 mg kg^−1^) and 2-methyl-3-nonene (0–0.26 mg kg^−1^) were cultivar-specific aroma substances, exclusive to ‘Tieton’ and ‘Nanyo’, respectively. Among these two unique terpenes, 5-methyl-1-heptene was primarily detected in the late stage of storage. Additionally, 6-methyl-1-heptene was identified as another distinct aromatic terpenoid compound specific to ‘Nanyo’.

In addition, ketones and acetic acids, though accounting for the lowest proportion of total VOCs, contributed unique olfactory attributes to cherries. Among ketones, m-Ethylacetophenone (with a relative concentration range of 0–1.97 mg kg^−1^) was the primary compound with a distinctive hawthorn flavor. In contrast, 1-Octen-3-one was well-known as an off-flavours in fruit derived from oxidative mechanisms. 3-Methyl-4-heptanone (0–0.08 mg kg^−1^) and 2-Octanone (0–1.48 mg kg^−1^) were detected across all cultivars, which marks their first identification in cherries, as they have previously only been reported in strawberries and kiwifruits [39,40], imparting soap and mushroom, pungent, fruity, earthy flavors to cherries, respectively. Additionally, acetyloxy-acetic acid (0–1.43 mg kg^−1^) was only detected in the early storage period of ‘Skeena’, giving cherries a sour taste. In comparison, 4-oxo-pentanoic acid (0–0.58 mg kg^−1^) was exclusive to ‘Tieton’ and ‘Nanyo’, imparting a distinctive apple-like flavor. Differently, hexadecenoic acid was found solely in ‘Nanyo’ and conferred an unpleasant taste. Generally, the presence of such acidic compounds affected cherry taste, often leading to the development of bitter, astringent, or salty notes. The low concentrations of ketones and acids may be attributed to the fact that acids and ketones are easily converted to alcohols, aldehydes, and esters via various enzyme systems [16].

Aroma research has demonstrated that the interactions among aroma components affect the final odor perception via synergistic or inhibitory effects. Specifically, the cherries contained relatively high levels of total volatile compounds, especially alcohols, aldehydes, and esters, all of which exert a significant impact on the fruit’s overall aroma profile. Among the alcohol compounds, E-2-hexen-1-ol and 2-ethyl-1-hexanol may be important factor in promoting the herbaceous flavor of cherries. For aldehydes, five of the identified aldehydes have been characterized as imparting green, grassy, or herbaceous (Table S3), including hexanal and E-2-hexenal; these aldehydes are therefore hypothesized to be at least partially responsible for the herbaceous off-flavor occasionally observed in sweet cherries. In contrast, the appearance of esters is associated with reduced aldehyde levels and the development of fruity flavor. Maybe esters could have an effect by inhibiting the perception of C6 aldehyde, thereby reducing the herbaceous flavor of cherries. Among the esters identified, 3-Methyl-1-butanol, acetate, and acetic acid, 2-ethylhexyl ester were the major contributors to the fruity flavor, especially for ‘Skeena’ cherries.

### 3.4. Analysis of Polyphenolic Compounds of Different Cherry Cultivars

Polyphenolic compounds exhibited great significance, because their content is directly associated with antioxidant capacity and health properties shown by cherries [27]. Cherries are rich in polyphenols, with more than 19 polyphenolic constituents detected, including phenolic acids and flavonoids. Specifically, a total of 7 phenolic acids and 13 flavonoids (anthocyanins (8), flavanols (2), flavonols (2), and flavanones (1)) were identified in the tested sweet cherries, a polyphenolic profile consistent with that reported in other sweet cherry cultivars [41]. Additionally, the content of polyphenols in plants displayed an important effect on the flavor quality of sweet cherry, which can be used as the main index to evaluate the flavor quality of cherry fruit.

Cultivar-specific variations in the composition and concentration of polyphenol compounds were disclosed in Table 2. Notably, neochlorogenic acid and quinic acid were identified as the primary phenolic acids, and this finding is consistent with observations from previous studies [37]. Quinic acid level was most abundant in ‘Benitemari’, where it occurred not only as a monomer but also in substantial quantities as complexes, such as caffeoylquinic acid and coumarylquinic acid. Among these conjugated forms, caffeoylquinic acid is recognized as the predominant quinic acid derivative in plants [15]. On the other hand, neochlorogenic acid, also known as 5-caffeoylquinic acid, was the main derivative of hydroxycinnamic acid; its content decreased during storage, a trend that paralleled the changes in total phenolic content. Chlorogenic acid belongs to phenolic acids generated by caffeic acid and quinic acid, namely caffeoylquinic acid, and followed a similar content variation pattern to neochlorogenic acid. Additionally, while differences in the content of these phenolic acids among cultivars were minimal, notable fluctuations were observed with increasing storage time. Obviously, the content of quinic acid increased during the late storage period, while chlorogenic acid levels decreased. Since one of the important raw materials for the synthesis of chlorogenic acid was quinic acid, it was speculated that the activity of transesterification enzymes with quinic acid decreased [42], or related expression genes were down-regulated, resulting in slower chlorogenic acid synthesis and increased quinic acid accumulation in the late storage period.

In addition, caffeic acid (0.50–18.81 mg kg^−1^), ferulic acid (0.72–8.22 mg kg^−1^), p-coumaric acid (0.12–10.38 mg kg^−1^), and vanillic acid (7.38–42.26 mg kg^−1^) were also detected. Notably, the content of these phenolic acids exhibited minimal variation among different cultivars. These phenolic acids have been reported to be associated with color, sensory, and nutritional qualities, as well as playing an important role in fruit ripening and the prevention of enzymatic browning [28]. In detail, the overall concentration of hydroxycinnamic acid and hydroxybenzoic acid in ‘Benitemari’ and ‘Nanyo’ was elevated, correlating with their lighter fruit peel color. This correlation can be attributed to the fact that hydroxycinnamic acid and hydroxybenzoic acid are the primary colorless phenolic compounds in sweet cherries, and these compounds are prone to conversion into colored anthocyanins [41].

Among flavonols, catechin and epicatechin were the primarily identified compounds. Epicatechin had a notably higher content, with its maximum concentration reaching 896.33 mg·kg^−1^, whereas the catechin content ranged from 0.72 to 81.12 mg·kg^−1^ across different cultivars. Moreover, the highest level of catechin was identified in ‘Benitemari’, while the epicatechin was most abundant in ‘Skeena’. Interestingly, the content of epicatechin consistently exceeded that of catechin, similar results had been reported in previous studies [23]. Catechins (including catechin and epicatechin) are widely recognized to contribute to the bitter taste of cherries, a sensory attribute also linked to catechins in lingonberry fruit [43].

For flavan-3-ols, rutin was mainly detected and known for their significant antioxidant and anti-inflammatory activities [44]. The content ranged from 24.33 to 408.60 mg kg^−1^, and this variation was strongly associated with cultivar genotype and fruit maturity, with higher rutin levels observed in black cherries. This finding aligns with the results of previous studies. Notably, kaempferol, another flavan-3-ol compound, was not detected in any of the analyzed sweet cherry samples.

Among flavanones, only naringenin was detected in the tested cherries. It primarily exists in the form of aglyins (namely naringin), and exhibits multiple beneficial biological activities, including potent anti-inflammatory effects, as well as anti-diabetic and anti-tumor properties [45]. However, the content of naringenin was minimal in cherries, with concentrations approximately 0.10 mg kg^−1^. Furthermore, it was hardly detected in the ‘Nanyo’ and ‘Benitemari’ cultivars.

Notable variability in anthocyanin accumulation was observed among the tested sweet cherry cultivars (Table 2). Both cultivated and wild cherry accessions exhibit a broad color spectrum, transitioning from green at the juvenile stage to yellow, red, purple, and black at full maturity. This color variation is primarily attributed to differences in the composition and concentration of anthocyanin fractions in the fruit peel.

However, anthocyanin is inherently destabilized and prone to degradation, and the most common stabilized formation is the 3-O-glucose bonded derivatives [10]. At present study, a total of nine anthocyanins were identified across the four cultivars, including cyanidin-3-O-glucoside (Cya-3-O-glu), cyanidin-3-O-rutinoside (Cya-3-O-rut), cyanidin-3-O-sophoroside (Cya-3-O-sop), delphinidin-3-O-rutinoside (Del-3-O-rut), pelargonidin-3-O-glucoside (Pel-3-O-glu), pelargonidin-3-O-rutinoside (Pel-3-O-rut), peonidin-3-O-glucoside (Peo-3-O-glu), peonidin-3-O-rutinoside (Peo-3-O-rut). The presence of these anthocyanins in sweet cherries was in accordance with other studies [1,15]. Furthermore, a strong positive correlation was observed between fruit color intensity and anthocyanin content. For instance, the red-black cultivars (‘Tieton’ and ‘Skeena’) were particularly rich in anthocyanins, with ‘Skeena’ showing particularly elevated concentrations of Cya-3-O-rut. Conversely, the light color cultivars, ‘Benitemari’ and ‘Nanyo’, were the poorest ones in anthocyanins, ranging from 0.00 to 1.26 mg kg-1. As reported in the previous literature, anthocyanins were virtually absent in yellow cherries [1]. Additionally, Cya-3-O-glu was identified as the primary anthocyanin in cherries, accounting for a large portion of the total anthocyanin content. Collectively, these findings emphasize that the composition, concentration, and relative proportions of anthocyanins play a critical role in determining sweet cherry fruit color, mirroring trends observed in other sweet and tart cherries [15,46].

**Figure 5:**
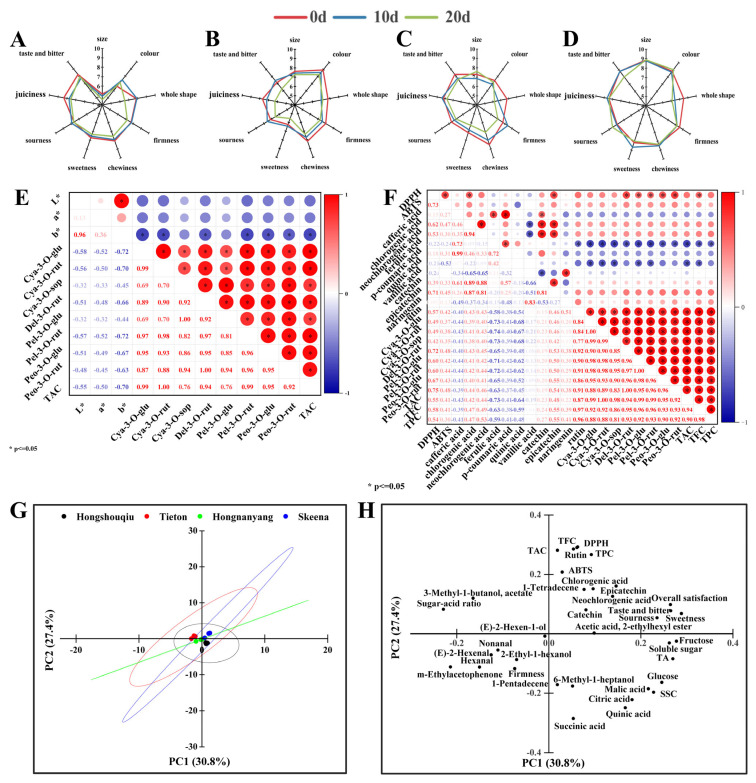
Sensory evaluation score radar map of ‘Benitemari’ (**A**), ‘Tieton’ (**B**), ‘Nanyo’ (**C**), ‘Skeena’ (**D**), Correlation analysis of color and anthocyanin content (**E**), Antioxidant activity and polyphenols (**F**), Score plot of cherries samples (**G**), Loading of variables (**H**) by the dimensions 1 and 2 from Principal component analysis of four different cultivars of cherry during storage at 0 °C for 20 d.

From Table 2, Cya-3-O-rut was identified as the predominant anthocyanin in both black cherries (‘Tieton’ and ‘Skeena’) and red-yellow cherries (‘Benitemari’ and ‘Nanyo’), with higher concentrations observed in the black cherries. Furthermore, Cya-3-O-rut was the only anthocyanin detected in red-yellow cherries (‘Benitemari’ and ‘Nanyo’), aligning with findings from previous studies [1]. In contrast, Cya-3-O-sop and Pel-3-O-glu were also at high levels, constituting the primary anthocyanin components in ‘Skeena’. Differences in the metabolites accumulated between the anthocyanin and proanthocyanidin pathways may be responsible for the color variation between yellow and black-red fruit. During fruit maturity, the progressive accumulation of anthocyanins is the key factor driving the development of the typical red-purple hue in cherries. This relationship between anthocyanin content and fruit color was further supported by the correlation analysis (Figure 5E), total anthocyanin content exhibited a significant negative correlation (*p* ≤ 0.05) with the color parameters L* (lightness), a* (red-green axis), and b* (yellow-blue axis). Specifically, the b* value showed significant (*p* ≤ 0.05) negative correlation with Cya-3-O-glu, Cya-3-O-rut, Pel-3-O-rut, and total anthocyanin content (TAC), indicating the synthesis of these specific anthocyanins is closely associated with the reduction in yellow tones in cherry peel, thereby modulating the final fruit peel color [13].

In order to verify the relationship between polyphenol compounds and antioxidant properties, a correlation analysis was conducted on the contents of individual polyphenolics, TAC, TPC and TFC (Figure 5F). The analysis revealed a strong positive correlation (in red areas) between antioxidant capacity and the contents of flavan-3-ols and anthocyanins. Furthermore, it can be seen that TPC and TFC were regarded as reliable predictors of sweet cherry antioxidant activity (*p* ≤ 0.05), as well as rutin and anthocyanins. Notably, the antioxidant activity of anthocyanins has been well characterized in in vitro studies [10]. Additionally, the data demonstrated a significant positive correlation between the contents of catechin, epicatechin, chlorogenic acid and neochlorogenic acid (correlation coefficient: r = 0.81–0.89, *p* ≤ 0.05). This strong correlation suggests a high degree of coordination in the biosynthesis of these polyphenolic compounds, which is likely attributed to their shared metabolic precursors and involvement in closely interconnected metabolic pathways.

### 3.5. Sensory Evaluation of Consumers for Different Cherry Cultivars

The sensory evaluation (Figure 5A–D) of sweet cherries revealed that ‘Skeena’ achieved the highest sensory scores, demonstrating superior comprehensive quality compared to other cultivars. According to Table S1, ‘Skeena’ was larger than ‘Nanyo’. As shown in Figure 1A, the ‘Skeena’ cultivar exhibited a significantly higher size score, which further confirmed that consumers generally show a preference for larger-sized sweet cherries. Furthermore, sensory attributes such as size, color, overall shape, firmness, chewiness, sweetness, sourness, juiciness, and the taste and bitterness were affected by storage time. A general decline in sensory scores was observed over time due to senescence [8]. Among them, ‘Skeena’ performed the smallest decline and better endurance, which was mainly related to its excellent overall quality. Specifically, consumer evaluations revealed significant differences across six specific attributes: size, color, firmness, sweetness, sourness, and taste profile (including bitterness), as thoroughly discussed in the previous sections.

Larger cherries were more appealing to consumers, as they were commonly perceived as more visually attractive, flavorful, and rich in pulp content [29]. Consistently, the black cherry cultivars (‘Tieton’ and ‘Skeena’) received higher color scores compared to the lighter-colored red-yellow cultivars (‘Benitemari’ and ‘Nanyo’). This observation indicates that consumers prefer red-black, glossy cherries—a preference likely driven by the widespread perception that deep fruit color is associated with health benefits and enhanced antioxidant properties, which is further supported by the results presented earlier (Figure 1 and Figure 2). Among all cultivars, ‘Skeena’ performed a higher score than the other three cultivars in sweetness, as well as taste and bitter, and the worst taste was found in ‘Tieton’, with the appearance of bitterness at 20 d. Two potential mechanisms may explain this phenomenon: in the early storage stage, sufficient sweet and sour tastes could mask the inherent bitterness of the fruit, leading to undetectable bitterness; in the late storage stage, however, the accumulation of bitter-tasting compounds (e.g., limonin and L-phenylpropionylglycine) may have intensified the perceived bitterness [13]. Samples with a sensory evaluation score above 8 were classified as of excellent quality. As shown in Figure 1 and Figure 5, the sweetness scores were greater than 8 corresponds to a mean SSC of over 18%, which is consistent with findings from previous studies [31]. Sourness scores above 8 corresponded to titratable acidity levels ranging from 0.50% to 0.56%. Obviously, this result was also within the scope of the previous literature, but on a much smaller scale. For firmness, the range of scores above 8 fell between 15.00 N and 17.38 N, which diverged from the literature due to the type of firmness testers and the expression method of firmness units [47].

### 3.6. Evaluation Model Results

#### 3.6.1. Relationships Between Sensory Evaluation and Cherry Characterizations

Different physicochemical attributes contribute varying degrees to consumer satisfaction with sweet cherries, and this satisfaction is predominantly influenced by key sensory traits including taste, juiciness, texture, and appearance [12]. These sensory traits are closely associated with parameters such as sugar content, organic acids, moisture levels, firmness, size, and color. To further identify the primary quality attribute influencing consumer satisfaction, a PCA was performed on the comprehensive multi-dimensional dataset generated in this study. The first principal component (PC1) explained 30.8% of the total variance of the original data. According to the principal component factors (Table 3), the PC1 was mainly based on sweetness score, sourness score, taste and bitter score, overall satisfaction score, SSC, soluble sugar, TA, malic acid, sugar-acid ratio, glucose, fructose, catechin, neochlorogenic acid, E-2-hexen-1-ol, hexanal, E-2-hexenal, nonanal, 3-methyl-1-butanol, acetate, acetic acid, 2-ethylhexyl ester, m-Ethylacetophenone. The second principal component (PC2) accounted for 27.4% of the coefficient of variation, with its variation mainly explained by attributes related to fruit texture and antioxidant capacity, including attributes of firmness, citric acid, succinic acid, quinic acid, chlorogenic acid, epicatechin, rutin, TPC, TFC, TAC, DPPH, ABTS, 2-ethyl-1-hexanol, 1-pentadecene, 1-tetradecene, 6-methyl-1-heptanol. These findings underscore the complex interrelationship between physiological quality and sensory evaluation in determining consumer satisfaction.

From Figure 5G,H, ‘Skeena’ cherry was located in the first quadrant; overall satisfaction, sweetness, sourness, and taste and bitter scores in the sensory evaluation were also in the first quadrant. This positioning indicated that ‘Skeena’ exhibited a higher trend of consumer satisfaction, elevated levels of sweetness and sourness scores, superior taste, and bitter scores. Similarly, the chemical reaction results, such as the radical scavenging rates of DPPH and ABTS, were also in the first quadrant, which also indicated that ‘Skeena’ cherry showed relatively high antioxidant properties. Conversely, ‘Nanyo’ was located in the third and fourth quadrants, characterized by a high relative concentration of VOCs of E-2-hexenal and hexanal, but exhibited low antioxidant activity, confirmed by the previous data.

From Table S2, fructose and malic acid emerged as major contributions to sweetness and acidity scores, respectively, suggesting that higher sweetness and acidity scores could improve consumer satisfaction. The taste and bitterness scores of cherries were primarily influenced by E-2-hexen-1-ol and acetic acid, 2-ethylhexyl ester, which endowed cherries with acetic acid and fruit flavors. It is worth noting that (E)-2-hexen-1-ol showed a negative correlation with taste and bitterness scores, suggesting that increased levels of this compound led to lower consumer satisfaction. Conversely, higher concentrations of 2-ethylhexyl acetate (acetic acid, 2-ethylhexyl ester) were linked to higher consumer satisfaction. Polyphenolic compounds, such as chlorogenic acid and neochlorogenic acid, were closer to the overall satisfaction and the taste and bitter scores, indicating that polyphenolics could improve taste and bitter scores. However, it was reported that polyphenolics could contribute to bitter flavors that negatively impact sensory evaluation; here, it may be due to the more adequate sweetness of ‘Skeena’ cherries that masked the bitter flavor [48,49]. Conversely, DPPH radical scavenging activity, ABTS radical scavenging activity, TAC (total anthocyanin content), TPC (total phenolic content), TFC (total flavonoid content), rutin, catechin, epicatechin, chlorogenic acid, and neochlorogenic acid were clustered together in the top part of the principal component analysis plot. This suggests that the free radical scavenging activities of DPPH and ABTS were associated with TAC, TPC, TFC, rutin, and other relevant substances. In addition, Del-3-O-rut, as an anthocyanin, exhibited the strongest correlation with the antioxidant activity (Figure 5C).

It is worth noting that significant differences exist in the postharvest storage characteristics of domestic and foreign sweet cherry cultivars. Based on the dynamic changes in quality indicators (SSC, firmness, peel color, and VOCs) during storage, combined with sensory evaluation results, the optimal storage conditions for different cultivars have been further clarified. Owing to geographical constraints, sweet cherries are generally not harvested at full ripeness; instead, they are picked once reaching commercial maturity, then immediately transported to cold storage for preservation or directly sold in the market.

For the foreign cultivar ‘Skeena’, the optimal storage condition was refrigeration at 0 °C for 10–20 days. This temperature effectively inhibited the degradation of the key aroma components of acetic acid and 2-ethylhexyl ester, thereby maintaining the unique flavor of the cherries, while also slowing down the loss of firmness. After 20 days of storage, the firmness remained at 15.20–16.80 N, and the SSC was still above 18%. The optimal storage condition for the domestic cultivar ‘Tieton’ is refrigeration at 0 °C for 10 days. ‘Tieton’ exhibited higher sensitivity to low temperatures than ‘Skeena’: when stored at 0 °C for more than 10 days, the content of cyanidin-3-O-rutinoside decreased by 15–20%, and the peel showed significant browning. Within 10 days of storage, ‘Tieton’ maintained stable quality, with SSC remaining at 18.2–19.5%, TA content at 0.52–0.55% (within the appropriate acidity range), and antioxidant activity retained at 80% of the initial level. When stored for more than 10 days, ‘Tieton’ showed a sharp decline in sensory scores due to an increase in titratable acid content. For ‘Benitemari’, considering its relatively low soluble sugar content compared to its SSC, the optimal storage condition is refrigeration at 0–1 °C for 10 days. This temperature effectively balanced the retention of SSC and the inhibition of organic acid accumulation, avoiding excessive sourness caused by increased acid content. When storage time exceeded 10 days, the flesh of ‘Benitemari’ softened, and its SSC dropped to less than 17.5%. Additionally, the light-colored sweet cherry cultivar ‘Nanyo’ was not suitable for long-term storage. It exhibited optimal sensory quality only when consumed immediately after harvest.

#### 3.6.2. The Establishment of the PCA Model

According to the above PCA results, the PC load was processed by SPSS software (Table S4), and the equation between PC and the basic component indicators of cherries was constructed as follows:

F1 = 0.960 × N1 + 0.783 × N2 + 0.882 × N3 + 0.880 × N4 − 0.246 × N5 + 0.758 × N6 + 0.872 × N7 + 0.898 × N8 + 0.603 × N9 + 0.720 × N10 + 0.177 × N11 − 0.763 × N12 + 0.818 × N13 + 0.923 × N14 + 0.308 × N15 + 0.203 × N16 + 0.209 × N17 + 0.097 × N18 + 0.063 × N19 + 0.269 × N20 + 0.321 × N21 + 0.554 × N22 + 0.178 × N23 + 0.462 × N24 + 0.487 × N25 − 0.028 × N26 − 0.232 × N27 − 0.501 × N28 − 0.416 × N29 − 0.368 × N30 − 0.548 × N31 − 0.711 × N32 + 0.062 × N33 + 0.255 × N34 + 0.328 × N35 + 0.172 × N36(1)

F2 = 0.214 × N1 + 0.169 × N2 + 0.244 × N3 + 0.309 × N4 − 0.369 × N5 − 0.618 × N6 − 0.096 × N7 − 0.265 × N8 − 0.698 × N9 − 0.582 × N10 − 0.896 × N11 + 0.260 × N12 − 0.514 × N13 − 0.078 × N14 + 0.838 × N15 + 0.912 × N16 + 0.920 × N17 + 0.653 × N18 + 0.884 × N19 + 0.253 × N20 + 0.476 × N21 − 0.783 × N22 + 0.896 × N23 + 0.397 × N24 + 0.506 × N25 − 0.025 × N26 − 0.274 × N27 − 0.353 × N28 − 0.223 × N29 − 0.172 × N30 + 0.377 × N31 − 0.351 × N32 − 0.538 × N33 + 0.469 × N34 + 0.008 × N35 − 0.553 × N36(2)

where N1–N36 represented sweetness score, sourness score, taste and bitter score, overall satisfaction score, firmness, SSC, soluble sugar, TA, citric acid, malic acid, succinic acid, sugar-acid ratio, glucose, fructose, TPC, TFC, DPPH, ABTS, TAC, catechin, chlorogenic acid, quinic acid, rutin, neochlorogenic acid, epicatechin, (E)-2-hexen-1-ol, 2-ethyl-1-hexanol, hexanal, (E)-2-hexenal, nonanal, 3-methyl-1-butanol, m-Ethylacetophenone, 1-pentadecene, 1-tetradecene, acetate, acetic acid and 6-methyl-1-heptanol, respectively.

Based on the principal component calculation formula, the model equation for the basic component content of BN was established as follows:

(3) Comprehensive score=0.53F1+0.47F2

The quality of sweet cherries should not be graded merely based on their appearance and size. Instead, exploring the intrinsic correlation between the comprehensive score and sensory evaluation scores is expected to reduce or eliminate the overreliance on appearance and size in cherry quality grading. Pearson correlation analysis indicated that the comprehensive score obtained by PCA was significantly positive correlated with the sweetness score and taste and bitter score in sensory evaluation (Table 4). Additionally, PCA exhibited a highly positive correlation with the firmness score, chewiness score, sourness score and juiciness score. However, no significant correlation was observed between the comprehensive score and either the size score or color score. This result indicates that cherry size and appearance color are not the sole determinants of sweet cherry quality. Furthermore, a positive trend was observed, with higher comprehensive scores corresponding to greater overall consumer acceptance of the cherries.

## 4. Conclusions

The present work was the first study focusing on the physical and chemical properties of domestic and foreign sweet cherries, focusing on the relationship between storage changes and consumer preferences. In aroma, the primary VOCs of different cultivars were (E)-2-hexenal and hexanal, which directly modulate the fresh sensory attribute of cherries. Additionally, acetic acid, 2-ethylhexyle ester may be a potential key aroma component of cherry flavor. Additionally, correlation analysis revealed a strong dependence of antioxidant activity and peel color, with cyanidin-3-O-rutinoside identified as the predominant anthocyanin. Furthermore, PCA and sensory evaluation score radar map suggested that the ideal sweet cherry was characterized by a high level of sweetness (SSC ≥ 18%), appropriate acidity (titratable acid content: 0.50–0.56%), suitable firmness (15.00–17.38 N) and a rich cherry flavor, influenced by acetic acid, 2-ethylhexyl ester. Among the cultivars tested, ‘Skeena’ performed the highest level of overall consumer satisfaction. In summary, significant variability in postharvest storage characteristics was observed between domestic and foreign cherry cultivars; this variability is largely attributed to genetic differences, as well as variations in climatic and soil conditions across cultivation regions. This study is expected to provide directions for the cultivation of high-quality cherry varieties from the perspective of consumer acceptance. Further research is necessary to reduce the sampling interval to better track the dynamic ripening trajectories of different cultivars and explore the relationship between polyphenol contents and the bitterness and taste profile of cherries.

## Figures and Tables

**Table 1:** Changes in antioxidant property in four different cultivars of cherry during storage at 0 °C for 20 days.

Radical Scavenging Activity (%)		DPPH	ABTS
**Benitemari**	0 d	27.02 ± 0.25 ^g^	76.36 ± 1.73 ^d^
	10 d	25.95 ± 1.14 ^h^	76.16 ± 4.91 ^d^
	20 d	19.98 ± 0.37 ^i^	51.47 ± 3.03 ^f^
**Tieton**	0 d	55.07 ± 3.14 ^c^	93.10 ± 0.88 ^a^
	10 d	61.78 ± 3.18 ^b^	92.80 ± 0.68 ^a^
	20 d	43.17 ± 1.10 ^d^	82.63 ± 2.96 ^c^
**Nanyo**	0 d	29.49 ± 0.24 ^f^	44.63 ± 1.02 ^g^
	10 d	27.97 ± 2.53 ^g^	57.21 ± 2.06 ^e^
	20 d	19.55 ± 0.24 ^i^	32.84 ± 0.70 ^h^
**Skeena**	0 d	92.47 ± 1.19 ^a^	94.30 ± 0.18 ^a^
	10 d	91.41 ± 0.52 ^a^	93.80 ± 0.36 ^a^
	20 d	35.91 ± 1.00 ^e^	89.16 ± 2.38 ^b^

Multiple comparisons by column, marked with different lowercase letters indicate significant differences between groups (*p* < 0.05).

**Table 2:** Polyphenolics compounds in four different cultivars of cherry during storage at 0 °C for 20 d.

Polyphenolics (mg kg^−1^)	Benitemari			Tieton			Nanyo			Skeena		
	0 d	10 d	20 d	0 d	10 d	20 d	0 d	10 d	20 d	0 d	10 d	20 d
**Phenolic acids**												
caffeic acid	1.73 ± 0.10 ^f^	8.26 ± 0.57 ^c^	3.03 ± 0.18 ^d^	1.45 ± 0.15 ^g^	2.48 ± 0.12 ^e^	1.67 ± 0.26 ^f^	18.81 ± 0.41 ^a^	16.45 ± 0.39 ^b^	0.86 ± 0.02 ^i^	1.27 ± 0.05 ^h^	1.44 ± 0.06 ^g^	0.50 ± 0.03 ^j^
chlorogenic acid	70.31 ± 3.59 ^g^	218.25 ± 11.46 ^c^	40.34 ± 0.68 ^h^	150.32 ± 20.24 ^f^	156.00 ± 9.66 ^ef^	42.33 ± 7.13 ^h^	172.79 ± 3.67 ^de^	255.18 ± 3.16 ^b^	10.29 ± 0.08 ^j^	189.52 ± 3.27 ^d^	288.63 ± 10.87 ^a^	16.98 ± 0.29 ^i^
neochlorogenic acid	1210.92 ± 58.17 ^e^	2020.55 ± 62.41 ^a^	805.88 ± 13.92 ^f^	1450.60 ± 159.36 ^d^	1395.1 ± 75.68 ^d^	554.53 ± 65.54 ^g^	1460.33 ± 15.91 ^d^	1665.79 ± 7.72 ^c^	251.1 ± 1.89 ^h^	1804.97 ± 7.10 ^b^	2051.31 ± 55.85 ^a^	554.9 ± 13.93 ^g^
ferulic acid	3.97 ± 0.21 ^e^	4.42 ± 0.09 ^d^	7.4 ± 0.17 ^b^	0.72 ± 0.07 ^g^	2.62 ± 0.14 ^e^	5.37 ± 0.93 ^c^	8.22 ± 0.06 ^a^	7.65 ± 0.19 ^a^	2.38 ± 0.03 ^e^	1.99 ± 0.01 ^g^	1.16 ± 0.04 ^g^	4.21 ± 0.12 ^f^
p-coumaric acid	0.54 ± 0.06 ^e^	3.5 ± 0.28 ^b^	1.35 ± 0.12 ^c^	0.75 ± 0.11 ^d^	1.03 ± 0.17 ^c^	0.65 ± 0.16 ^de^	10.38 ± 0.23 ^a^	10.2 ± 0.32 ^a^	0.29 ± 0.01 ^f^	0.27 ± 0.04 ^f^	0.72 ± 0.05 ^d^	0.12 ± 0.01 ^g^
quinic acid	1133.44 ± 37.81 ^a^	940.71 ± 15.68 ^c^	1036.31 ± 13.44 ^b^	256.43 ± 33.92 ^i^	422.92 ± 16.87 ^h^	418.15 ± 53.56 ^h^	508.02 ± 3.24 ^g^	803.82 ± 25.16 ^d^	708.48 ± 14.33 ^e^	549.13 ± 5.84 ^f^	509.02 ± 8.79 ^g^	721.72 ± 4.65 ^e^
vanillic acid	11.31 ± 0.69 ^f^	7.38 ± 0.24 ^g^	17.11 ± 0.35 ^c^	7.80 ± 1.05 ^g^	15.17 ± 1.55 ^cd^	22.86 ± 3.84 ^b^	11.48 ± 0.19 ^f^	8.61 ± 0.05 ^g^	14.47 ± 0.62 ^d^	13.12 ± 0.09 ^e^	8.36 ± 0.29 ^g^	42.26 ± 1.25 ^a^
**Flavanols**												
catechin	17.3 ± 0.88 ^gh^	81.12 ± 4.12 ^a^	15.62 ± 0.25 ^h^	50.08 ± 5.32 ^de^	34.26 ± 1.04 ^fg^	11.09 ± 1.75 ^i^	74.59 ± 1.87 ^b^	54.78 ± 1.71 ^d^	0.72 ± 0.03 ^k^	38.23 ± 0.36 ^ef^	69.76 ± 0.92 ^c^	2.40 ± 0.11 ^j^
epicatechin	59.06 ± 3.11 ^h^	433.16 ± 18.83 ^b^	45.04 ± 2.01 ^i^	206.89 ± 18.66 ^f^	120.43 ± 9.07 ^g^	25.92 ± 3.38 ^j^	382.08 ± 5.07 ^c^	306.51 ± 5.85 ^e^	3.65 ± 0.02 ^l^	328.07 ± 0.80 ^d^	896.33 ± 48.59 ^a^	19.38 ± 0.51 ^k^
**Flavonols**												
rutin	72.15 ± 4.28 ^f^	97.87 ± 9.59 ^e^	69.99 ± 1.48 ^f^	205.11 ± 22.9 ^c^	277.49 ± 15.59 ^b^	214.18 ± 30.55 ^c^	50.51 ± 2.12 ^g^	127.08 ± 1.94 ^d^	24.33 ± 0.16 ^h^	408.6 ± 12.41 ^a^	383.88 ± 6.56 ^a^	208.06 ± 6.84 ^c^
kaempferol	--	--	--	--	--	--	--	--	--	--	--	--
**Flavanones**												
naringenin	0.04 ± 0.01 ^gh^	0.03 ± 0.00 ^h^	0.08 ± 0.01 ^e^	--	0.11 ± 0.02 ^d^	0.19 ± 0.04 ^c^	--	0.02 ± 0.01 ^i^	0.05 ± 0.00 ^fg^	0.23 ± 0.01 ^b^	0.07 ± 0.01 ^ef^	0.32 ± 0.01 ^a^
**Anthocyanin**												
Cya-3-O-glu	--	--	--	24.36 ± 1.59 ^a^	8.00 ± 1.21 ^b^	8.94 ± 6.12 ^b^	--	--	--	22.85 ± 0.63 ^a^	23.49 ± 0.69 ^a^	4.24 ± 0.37 ^c^
Cya-3-O-rut	1.62 ± 0.30 ^e^	0.78 ± 0.02 ^f^	1.26 ± 0.08 ^e^	634.39 ± 62.39 ^a^	253.82 ± 26.58 ^b^	180.96 ± 89.54 ^c^	0.57 ± 0.34 ^f^	--	--	605.24 ± 17.32 ^a^	595.13 ± 32.52 ^a^	88.23 ± 1.18 ^d^
Cya-3-O-sop	--	--	--	1.62 ± 0.13 ^a^	0.44 ± 0.07 ^c^	0.36 ± 0.21 ^c^	--	--	--	1.25 ± 0.02 ^b^	1.21 ± 0.06 ^b^	0.18 ± 0.01 ^d^
Del-3-O-rut	--	--	--	1.89 ± 0.23 ^c^	1.20 ± 0.13 ^d^	0.61 ± 0.45 ^e^	--	--	--	4.72 ± 0.19 ^a^	3.85 ± 0.24 ^b^	1.03 ± 0.04 ^de^
Pel-3-O-glu	--	--	--	0.27 ± 0.02 ^b^	0.11 ± 0.01 ^c^	0.11 ± 0.06 ^c^	--	--	--	0.36 ± 0.00 ^a^	0.34 ± 0.02 ^a^	0.08 ± 0.01 ^d^
Pel-3-O-rut	--	--	--	7.44 ± 0.49 ^b^	3.93 ± 0.33 ^c^	2.48 ± 1.12 ^cd^	--	--	--	10.27 ± 0.36 ^a^	9.57 ± 0.56 ^a^	2.15 ± 0.14 ^d^
Peo-3-O-glu	--	--	--	0.22 ± 0.03 ^b^	0.02 ± 0.01 ^d^	0.09 ± 0.08 ^c^	--	--	--	0.33 ± 0.01 ^a^	0.34 ± 0.01 ^a^	0.09 ± 0.01 ^c^
Peo-3-O-rut	--	--	--	62.6 ± 1.70 ^c^	36.65 ± 2.9 ^d^	20.87 ± 12.61 ^e^	--	--	--	178.01 ± 1.8 ^a^	143.69 ± 7.70 ^b^	34.88 ± 1.73 ^d^

Multiple comparisons by row, marked with different lowercase letters indicate significant differences between groups (*p* < 0.05).

**Table 3:** Comprehensive score of principal components of fruit nutritional quality of four different cultivars of cherry during storage at 0 °C for 20 days.

Code		Principal Component		Comprehensive Score	Ranking
Cherry Cultivars	Storage Time	PC1	PC2		
Benitemari	0 d	0.90595	−1.21262	−0.0908	5
	10 d	0.60539	−1.07751	−0.18668	6
	20 d	0.6681	−1.40778	−0.30914	10
Tieton	0 d	−1.26542	0.79557	−0.29677	9
	10 d	−1.04924	0.66734	−0.24245	8
	20 d	−1.66258	0.01193	−0.87724	12
Nanyo	0 d	0.07898	−0.08674	0.001033	4
	10 d	−0.16268	−0.2371	−0.1982	7
	20 d	−0.93594	−0.73613	−0.84416	11
Skeena	0 d	1.08848	1.46221	1.267584	2
	10 d	1.25938	1.58379	1.415673	1
	20 d	0.46958	0.23706	0.361154	3

**Table 4:** Correlation between the comprehensive score of the principal component and the sensory evaluation score.

Sensory Evaluation Indicator	Correlation Coefficient
Size	0.5
Color	0.44
Whole shape	0.44
Firmness	0.76 *
Chewiness	0.79 *
Sweetness	0.86 **
Sourness	0.7 *
Juiciness	0.7 *
Taste and bitter	0.82 *

* *p* < 0.05; ** *p* < 0.001.

## Data Availability

The original contributions presented in this study are included in the article. Further inquiries can be directed to the corresponding authors.

## Supplementary Materials

The following supporting information can be downloaded at: https://www.mdpi.com/article/10.3390/foods14193432/s1, Table S1: The basic physical attributes in four different cultivars of cherry in this study; Table S2: Changes in Soluble sugar components and organic acid components in four different cultivars of cherry during storage at 0 °C for 20 d. Table S3: Volatile organic compounds in four different cultivars of cherry during storage at 0 °C for 20 d. Table S4: Eigenvalues, variance contribution rate, cumulative contribution rate and component loading moment of principal components.

## References

[1] Chu L., Zheng W., Wang J., Wang Z., Zhao W., Zhao B., Xu G.H., Xiao M., Lou X., Pan F.R. (2023). Comparative analysis of the difference in flavonoid metabolic pathway during coloring between red-yellow and red sweet cherry (*Prunus avium* L.). Gene.

[2] Acero N., Gradillas A., Beltran M., García A., Mingarro D.M. (2019). Comparison of phenolic compounds profile and antioxidant properties of different sweet cherry (*Prunus avium* L.) varieties. Food Chem..

[3] Jesus F., Goncalves A.C., Alves G., Silva L.R. (2022). Health benefits of *Prunus avium* plant parts: An unexplored source rich in phenolic compounds. Food Rev. Int..

[4] Zheng H.Y., Deng W.Q., Yu L., Shi Y.C., Deng Y., Wang D.F., Zhong Y. (2024). Chitosan coatings with different degrees of deacetylation regulate the postharvest quality of sweet cherry through internal metabolism. Int. J. Biol. Macromol..

[5] Meng X.Y., Chen C., Song T., Xu J.W., Zhang X.B., Wang J., Pan Z.L., Zhang H., Zhang H.J. (2022). Effect of nano-silica coating combined with pressurized Ar treatment on postharvest quality and reactive oxygen species metabolism in sweet cherry fruit. Food Chem..

[6] Pinto de Andrade L., Veloso A., Espírito Santo C., Dinis Gaspar P., Silva P.D., Resende M., Beato H., Baptista C., Pintado M.C., Paulo L. (2022). Effect of controlled atmospheres and environmental conditions on the physicochemical and sensory characteristics of sweet cherry cultivar Satin. Agronomy.

[7] Miranda S., Vilches P., Suazo M., Pavez L., García K., Méndez M.A., Meisel L.A., Defilippi B.G., Del Pozo T. (2020). Melatonin triggers metabolic and gene expression changes leading to improved quality traits of two sweet cherry cultivars during cold storage. Food Chem..

[8] Cozzolino R., Martignetti A., Cefola M., Pace B., Capotorto I., De Giulio B., Montemurro N., Pellicano M.P. (2019). Volatile metabolites, quality and sensory parameters of “Ferrovia” sweet cherry cold stored in air or packed in high CO_2_ modified atmospheres. Food Chem..

[9] Zhao H.D., Liu B.D., Zhang W.L., Cao J.K., Jiang W.B. (2019). Enhancement of quality and antioxidant metabolism of sweet cherry fruit by near-freezing temperature storage. Postharvest Biol. Technol..

[10] Sun L.P., Huo J.T., Liu J., Yu J.Y., Zhou J.L., Sun C.D., Wang Y., Leng F. (2023). Anthocyanins distribution, transcriptional regulation, epigenetic and post-translational modification in fruit. Food Chem..

[11] Zhang C.L., Gong H.S., Liu Y.L. (2022). Effects of postharvest coating using chitosan combined with natamycin on physicochemical and microbial properties of sweet cherry during cold storage. Int. J. Biol. Macromol..

[12] Silva V., Pereira S., Vilela A., Bacelar E., Guedes F., Ribeiro C., Silva A., Gonçalves B. (2021). Preliminary insights in sensory profile of sweet cherries. Foods.

[13] Liu Z.S., Wang H., Zhang J., Chen Q., He W., Zhang Y., Luo Y., Tang H.R., Wang Y., Wang X.R. (2024). Comparative metabolomics profiling highlights unique color variation and bitter taste formation of Chinese cherry fruit. Food Chem..

[14] López L., Larrigaudière C., Giné-Bordonaba J., Echeverria G. (2023). Defining key parameters and predictive markers of ‘Early Bigi’cherry consumer satisfaction by means of differential storage scenarios. Postharvest Biol. Technol..

[15] Karagiannis E., Sarrou E., Michailidis M., Tanou G., Ganopoulos I., Bazakos C., Kazantzis K., Martens S., Xanthopoulou A., Molassiotis A. (2021). Fruit quality trait discovery and metabolic profiling in sweet cherry genebank collection in Greece. Food Chem..

[16] Vavoura M.V., Badeka A.V., Kontakos S., Kontominas M.G. (2015). Characterization of four popular sweet cherry cultivars grown in Greece by volatile compound and physicochemical data analysis and sensory evaluation. Molecules.

[17] Zhang Y.Q., Pu Y.J., Jiang H.T., Chen L.Y., Shen C.Y., Zhang W.L., Cao J.K., Jiang W.B. (2024). Improved sustained-release properties of ginger essential oil in a Pickering emulsion system incorporated in sodium alginate film and delayed postharvest senescence of mango fruit. Food Chem..

[18] Zhang Y., Wang H., Chen H., Liu R., Chen H., Fang X., Xiao J., Wu W., Gao H. (2024). The crucial evaluation indexes and relative measurement methods of edible value for fresh fruits and vegetables: A review. Future Postharvest Food.

[19] Fan X.G., Zhao H.D., Wang X.M., Cao J.K., Jiang W.B. (2017). Sugar and organic acid composition of apricot and their contribution to sensory quality and consumer satisfaction. Sci. Hortic..

[20] Wang H.X., Zhang Y.P., Pu Y.J., Chen L.Y., He X., Cao J.K., Jiang W.B. (2023). Composite coating of guar gum with salicylic acid alleviates the quality deterioration of vibration damage in ‘Huangguan’ pear fruit through the regulation of antioxidant metabolism. Postharvest Biol. Technol..

[21] Panić M., Radić Stojković M., Kraljić K., Škevin D., Radojčić Redovniković I., Gaurina Srček V., Radošević K. (2019). Ready-to-use green polyphenolic extracts from food by-products. Food Chem..

[22] Dong F., Wang X. (2018). Guar gum and ginseng extract coatings maintain the quality of sweet cherry. LWT-Food Sci. Technol..

[23] Gonçalves A.C., Campos G., Alves G., Garcia-Viguera C., Moreno D.A., Silva L.R. (2021). Physical and phytochemical composition of 23 Portuguese sweet cherries as conditioned by variety (or genotype). Food Chem..

[24] Adhikary T., Gill P.P.S., Jawandha S.K., Sinha A. (2022). Chitosan coating modulates cell wall degrading enzymes and preserved postharvest quality in cold-stored pear fruit. J. Food Meas. Charact..

[25] Zhang A.D., Yang H.Y., Ji S.J., Tian C.P., Chen N., Gong H.S., Li J.Z. (2022). Metabolome and transcriptome analyses of anthocyanin accumulation mechanisms reveal metabolite variations and key candidate genes involved in the pigmentation of *Prunus tomentosa* Thunb. cherry fruit. Front. Plant Sci..

[26] Zhang J., Wang Y., Chen T., Chen Q., Wang L., Liu Z.S., Wang H., Xie R., He W., Li M. (2021). Evolution of Rosaceae plastomes highlights unique Cerasus diversification and independent origins of fruiting cherry. Front. Plant Sci..

[27] Chockchaisawasdee S., Golding J.B., Vuong Q.V., Papoutsis K., Stathopoulos C.E. (2016). Sweet cherry: Composition, postharvest preservation, processing, and trends for its future use. Trends Food Sci. Technol..

[28] Chezanoglou E., Mourtzinos I., Goula A.M. (2024). Sweet cherry and its by-products as sources of valuable phenolic compounds. Trends Food Sci. Technol..

[29] Di Matteo A., Russo R., Graziani G., Ritieni A., Di Vaio C. (2017). Characterization of autochthonous sweet cherry cultivars (*Prunus avium* L.) of southern Italy for fruit quality, bioactive compounds and antioxidant activity. J. Sci. Food Agric..

[30] Zhang H.M., Tu K., Qiu Z.L., Wen Z., Li Q., Wen X. (2021). Effects of different rain shelter coverings on volatile organic compounds in mature fruit and postharvest quality of sweet cherry. CyTA-J. Food.

[31] Blando F., Oomah B.D. (2019). Sweet and sour cherries: Origin, distribution, nutritional composition and health benefits. Trends Food Sci. Technol..

[32] Michailidis M., Karagiannis E., Tanou G., Sarrou E., Karamanoli K., Lazaridou A., Martens S., Molassiotis A. (2020). Sweet cherry fruit cracking: Follow-up testing methods and cultivar-metabolic screening. Plant Methods.

[33] Zhou J.T., Yang S.W., Ma Y., Liu Z.S., Tu H.X., Wang H., Zhang J., Chen Q., He W., Li M.Y. (2023). Soluble sugar and organic acid composition and flavor evaluation of Chinese cherry fruit. Food Chem. X.

[34] Habib M., Bhat M., Dar B.N., Wani A.A. (2017). Sweet cherries from farm to table: A review. Crit. Rev. Food Sci. Nutr..

[35] Ma D., Zhao H.Y., Liu Z.Z., Liu M.Q., Qi P.P., Di S.S., Zhang S.L., Wang X.Q. (2023). Recent advances on mulberry volatile flavor: A review. J. Food Compos. Anal..

[36] Zhu J.C., Wang L.Y., Xiao Z.B., Niu Y.W. (2018). Characterization of the key aroma compounds in mulberry fruit by application of gas chromatography–olfactometry (GC-O), odor activity value (OAV), gas chromatography-mass spectrometry (GC–MS) and flame photometric detection (FPD). Food Chem..

[37] Hayaloglu A.A., Demir N. (2016). Phenolic compounds, volatiles, and sensory characteristics of twelve sweet cherry (*Prunus avium* L.) cultivars grown in Turkey. J. Food Sci..

[38] Luo M., Zhou X., Sun H., Zhou Q., Ge W., Sun Y., Yao M., Ji S. (2021). Insights into profiling of volatile ester and LOX-pathway related gene families accompanying post-harvest ripening of ‘Nanguo’ pears. Food Chem..

[39] Van de Poel B., Vandendriessche T., Hertog M.L., Nicolai B.M., Geeraerd A. (2014). Detached ripening of non-climacteric strawberry impairs aroma profile and fruit quality. Postharvest Biol. Technol..

[40] Han X.Y., Wang X.Y., Shen C., Mo Y.W., Tian R.G., Mao L.C., Luo Z.S., Yang H.Y. (2022). Exogenous ABA promotes aroma biosynthesis of postharvest kiwifruit after low-temperature storage. Planta.

[41] Gao Y., Wang M., Jiang N., Wang Y., Feng X.Y. (2019). Use of ultra-performance liquid chromatography–tandem mass spectrometry on sweet cherries to determine phenolic compounds in peel and flesh. J. Sci. Food Agric..

[42] Hoffmann L., Besseau S., Geoffroy P., Ritzenthaler C., Meyer D., Lapierre C., Pollet B., Legrand M. (2004). Silencing of hydroxycinnamoyl-coenzyme A shikimate/quinate hydroxycinnamoyl transferase affects phenylpropanoid biosynthesis. Plant Cell.

[43] Xu J., Li H.Q., Yang H., Wang T., Chang Y.H., Nie C.D., Nie S.M., Fu Y.J. (2024). Lingonberry (*Vaccinium vitis-idaea* L.) fruit: Potential characterization of flavor and functional profiles during ripening based on UHPLC-QqQ-MS/MS. J. Food Compos. Anal..

[44] Yong D.O., Saker S.R., Chellappan D.K., Madheswaran T., Panneerselvam J., Choudhury H., Pandey M., Chan Y.L., Collet T., Gupta G. (2020). Molecular and immunological mechanisms underlying the various pharmacological properties of the potent bioflavonoid, rutin. Endocr. Metab. Immune Disord.-Drug Targets.

[45] Csuti A., Sik B., Ajtony Z. (2022). Measurement of naringin from citrus fruit by high-performance liquid chromatography: A review. Crit. Rev. Anal. Chem..

[46] Homoki J.R., Nemes A., Fazekas E., Gyémánt G., Balogh P., Gál F., Al-Asri J., Mortier J., Wolber G., Babinszky L. (2016). Anthocyanin composition, antioxidant efficiency, and α-amylase inhibitor activity of different Hungarian sour cherry varieties (*Prunus cerasus* L.). Food Chem..

[47] Hampson C.R., Stanich K., McKenzie D.L., Herbert L., Lu R., Li J., Cliff M.A. (2014). Determining the optimum firmness for sweet cherries using Just-About-Right sensory methodology. Postharvest Biol. Technol..

[48] Bertelsen A.S., Laursen A., Knudsen T.A., Møller S., Kidmose U. (2018). Bitter taste masking of enzyme--treated soy protein in water and bread. J. Sci. Food Agric..

[49] Shi J., Nawaz H., Pohorly J., Mittal G., Kakuda Y., Jiang Y. (2005). Extraction of polyphenolics from plant material for functional foods—Engineering and technology. Food Rev. Int..

