# Supplementary material for: Integrative Comparison of Variations in Taste, Aroma, and Sensory Characteristics Among Four Sweet Cherry Cultivars to Explore Quality Differences During Storage
Source: Foods. 2025 Oct 7;14(19):3432. doi: 10.3390/foods14193432 (PMC12523664; doi:10.3390/foods14193432)
Supplement: Supplementary file 1 [file foods-14-03432-s001.zip › foods-3877949-supplementary.pdf]

Integrative comparison of variations in taste, aroma, and sensory characteristics among four sweet cherry cultivars to explore quality differences during storage

Table supplement

Table S1. The basic physical attributes in four different cultivars of cherry in this study.

Cultivar	Benitemari	Nanyo	Tieton	Skeena
**Appearance photo**	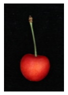	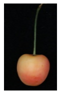	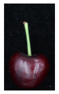	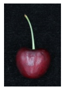
**Origin source**	Lvshun, China	Lvshun, China	Lvshun, China	Washington, U. S
**Weight (g)**	7.95 ± 0.79	11.23 ± 1.62	10.41 ± 1.79	12.71 ± 0.85
**Width (mm)**	24.31 ± 1.07	27.49 ± 1.54	26.80 ± 2.53	29.41 ± 1.84
**Height (mm)**	22.28 ± 0.97	22.28 ± 0.97	24.55 ± 1.50	25.46 ± 0.98
**Thickness (mm)**	21.18 ± 1.07	22.80 ± 1.30	22.91 ± 1.52	24.52 ± 1.05
**Soluble solid content (%)**	23.88±0.04	13.55±0.45	18.62±0.17	19.67±0.4
**Appearance**	Yellow-red	Yellow	Black-red	Black-red

The cultivar 'Tieton' is the most common on the Chinese market in summer, but cultivar 'Benitemari' and cultivar 'Nanyo' belong to light-colored cherries with less study. And cultivar 'Skeena' is also common on the Chinese market, and they were from Washington, U. S, where the climate is mild and humid all the year round, it is suitable for the growth of cherries with good cherry quality.

Table S2. Changes of Soluble sugar components and organic acid components in four different cultivars of cherry during storage at 0 ℃ for 20 d.

**Components**	**Benitemari**			**Tieton**			**Nanyo**			**Skeena**		
**(mg kg^{-1})**	**0 d**	**10 d**	**20 d**	**0 d**	**10 d**	**20 d**	**0 d**	**10 d**	**20 d**	**0 d**	**10 d**	**20 d**
**Soluble sugar**												
Glucose sugar	125.10 ± 6.90^{a}	122.20 ± 5.73^{a}	118.63 ± 1.62^{a}	52.43 ± 0.21^{d}	58.83 ± 0.51^{d}	52.63 ± 0.47^{d}	72.77 ± 1.17^{c}	81.43 ± 1.27^{c}	73.07 ± 3.77^{c}	95.13 ± 1.31^{b}	90.00 ± 1.28^{b}	86.20 ± 0.60^{b}
Fructose sugar	53.17 ± 2.71^{a}	51.43 ± 2.57^{ab}	49.57 ± 0.8^{ab}	31.33 ± 0.21^{e}	37.17 ± 0.21^{d}	36.13 ± 0.38^{d}	39.60 ± 0.62^{cd}	42.33 ± 0.58^{bc}	41.47 ± 2.31^{bc}	55.07 ± 1.12^{a}	52.80 ± 0.40^{a}	49.57 ± 0.80^{ab}
Sucrose sugar	0.40 ± 0.00^{a}	0.43 ± 0.06^{a}	0.40 ± 0.00^{a}	0.47 ± 0.06^{a}	0.43 ± 0.06^{a}	0.43 ± 0.06^{a}	0.47 ± 0.06^{a}	0.40 ± 0.00^{a}	0.43 ± 0.06^{a}	0.40 ± 0.00^{a}	0.43 ± 0.06^{a}	0.47 ± 0.06^{a}
Maltose sugar	0.80 ± 0.00^{b}	0.93 ± 0.06^{a}	0.90 ± 0.00^{a}	0.47 ± 0.06^{d}	0.63 ± 0.06^{c}	0.67 ± 0.12^{c}	0.50 ± 0.20^{d}	0.77 ± 0.21^{b}	0.67 ± 0.12^{c}	0.53 ± 0.06^{d}	0.63 ± 0.06^{c}	0.53 ± 0.06^{d}
Lactose sugar	0.03 ± 0.00^{a}	0.03 ± 0.00^{a}	0.03 ± 0.06^{a}	0.01 ± 0.00^{c}	0.01 ± 0.00^{c}	0.01 ± 0.00^{c}	0.01 ± 0.01^{d}	0.02 ± 0.00^{d}	0.02 ± 0.00^{d}	0.02 ± 0.00^{d}	0.02 ± 0.00^{d}	0.02 ± 0.00^{d}
Total content	180.50 ± 7.42^{a}	175.02 ± 6.26^{a}	169.53 ± 1.82^{a}	84.69 ± 0.41^{d}	97.07 ± 0.59^{c}	90.00 ± 0.65^{c}	113.35 ± 1.35^{c}	124.95 ± 1.39^{b}	115.66 ± 4.43^{b}	151.15 ± 1.73^{b}	143.88 ± 1.36^{b}	136.79 ± 1.05^{b}
**Organic acid**												
Malic acid	20.79 ± 0.15^{a}	19.42 ± 0.09^{b}	20.21 ± 0.19^{a}	9.45 ± 0.17^{f}	8.72 ± 0.12^{f}	8.82 ± 0.11^{f}	10.95 ± 0.26^{e}	10.69 ± 0.11^{e}	10.64 ± 0.16^{e}	13.40 ± 0.09^{d}	13.50 ± 0.08^{d}	13.00 ± 0.72^{d}
Succinic acid	2.71 ± 0.03^{a}	2.87 ± 0.02^{a}	2.57 ± 0.03^{a}	1.54 ± 0.03^{d}	1.87 ± 0.04^{c}	1.93 ± 0.02^{c}	1.98 ± 0.05^{c}	2.28 ± 0.04^{b}	2.24 ± 0.04^{b}	1.58 ± 0.01^{d}	1.59 ± 0.03^{d}	1.54 ± 0.08^{d}
Citric acid	1.71 ± 0.01^{a}	1.61 ± 0.00^{b}	1.42 ± 0.02^{c}	0.43 ± 0.00^{f}	0.41 ± 0.00^{f}	0.58 ± 0.01^{e}	0.74 ± 0.02^{d}	0.81 ± 0.02^{d}	0.61 ± 0.01^{e}	0.77 ± 0.01^{d}	0.74 ± 0.01^{d}	0.65 ± 0.03^{e}
Tartaric acid	0.15 ± 0.00^{d}	0.17 ± 0.00^{c}	0.20 ± 0.00^{b}	0.29 ± 0.01^{a}	0.26 ± 0.00^{a}	0.28 ± 0.01^{a}	0.20 ± 0.00^{b}	0.26 ± 0.01^{a}	0.28 ± 0.01^{a}	0.19 ± 0.00^{b}	0.22 ± 0.00^{b}	0.21 ± 0.01^{b}
Fumaric acid	0.002 ± 0.00^{c}	0.001 ± 0.00^{d}	0.001 ± 0.00^{d}	0.003 ± 0.00^{c}	0.005 ± 0.00^{b}	0.009 ± 0.00^{a}	0.002 ± 0.00^{c}	0.004 ± 0.00^{b}	0.006 ± 0.00^{b}	0.000 ± 0.00^{e}	0.000 ± 0.00^{e}	0.003 ± 0.00^{c}
Total content	25.36 ± 0.16^{a}	24.07 ± 0.10^{b}	24.40 ± 0.20^{b}	11.71 ± 0.18^{f}	11.27 ± 0.13^{f}	11.62 ± 0.12^{f}	13.87 ± 0.27^{e}	14.04 ± 0.13^{e}	13.78 ± 0.17^{e}	15.94 ± 0.10^{d}	16.05 ± 0.10^{d}	15.40 ± 0.73^{d}

Multiple comparisons by row, marked with different lowercase letters indicate significant differences between groups (p<0.05).

Table S3. Volatile organic compounds in four different cultivars of cherry during storage at 0 ℃ for 20 d.

VOCs (mg kg-1)	Benitemari			Tieton			Nanyo			Skeena			flavor
	0 d	10 d	20 d	0 d	10 d	20 d	0d	10d	20d	0d	10d	20d	
**Alcohols**													
2-Methyl-1-pentanol	0.17±0.01	0.26±0.02	0.19±0.01	0.20±0.01	--	--	0.22±0.06	--	--	--	--	--	
3-Methyl-1-pentanol	0.05±0.02	0.06±0.01	--	--	--	0.05±0.00	0.03±0.01	0.01±0.00	--	--	--	--	grape wine, cocoa, fruit
Cyclohexanol	--	4.90±0.22	--	--	--	--	2.99±0.13	--	--	--	--	--	camphor
(E)-2-hexen-1-ol	6.09±1.31	--	3.76±0.08	1.80±0.26	3.13±0.04	6.13±1.05	0.52±0.13	--	2.52±0.07	2.00±0.11	4.04±0.41	4.51±0.35	green, fruity, herbaceous, leaf
3-Methyl-1-hexanol	0.14±0.00	--	0.04±0.00	0.10±0.03	0.21±0.10	--	0.05±0.01	0.05±0.00	0.11±0.04	0.07±0.03	0.06±0.01	--	flower
4-Methyl-1-hexanol	--	--	0.06±0.01	0.14±0.05	--	0.06±0.03	--	0.05±0.02	--	--	0.03±0.00	--	floral, grape
2-Ethyl-1-hexanol	8.89±0.09	9.96±0.73	15.89±2.14	10.49±0.33	11.82±1.52	11.77±0.79	8.14±1.06	8.91±0.93	12.28±1.64	13.05±2.07	6.65±0.62	7.35±0.93	flower
1-Heptanol	0.16±0.07	--	0.20±0.05	0.17±0.03	0.19±0.11	0.40±0.16	0.30±0.08	0.35±0.13	0.44±0.17	0.19±0.08	0.24±0.01	0.43±0.22	green, dry, resin, flower
3-Heptanol	--	--	0.05±0.00	--	--	--	--	--	--	--	--	--	herbaceous
5-Methyl-1-heptanol	--	--	0.23±0.16	0.06±0.01	0.16±0.06	--	--	0.08±0.03	0.11±0.05	--	--	0.04±0.00	
6-Methyl-1-heptanol	0.52±0.10	0.19±0.03	0.43±0.15	0.18±0.09	0.28±0.12	0.11±0.04	0.40±0.21	0.18±0.06	0.06±0.02	0.07±0.00	0.09±0.02	--	
Benzyl alcohol	1.32±0.15	1.92±0.23	0.52±0.20	--	--	--	--	--	--	--	--	1.10±0.31	sweet, floral, fruity
1-Octanol	--	--	0.06±0.01	--	0.10±0.03	0.22±0.02	--	0.23±0.06	--	0.21±0.11	--	--	Sweet, floral, fruity, citrus
(S)-3,7-Dimethyl-7-octen-1-ol	--	--	--	--	--	0.12±0.01	--	--	0.13±0.04	--	--	0.30±0.13	pungent, fruity, earthy
2,7-Dimethyl-1-octanol	--	1.17±0.37	--	--	0.66±0.30	--	0.98±0.22	--	--	--	0.43±0.24	--	

1-Undecanol	--	--	--	--	--	0.78±0.21	--	0.14±0.05	0.42±0.10	0.56±0.09	--	--	rose、fruit、sweet
**Total Alcohol content**	17.35±1.33	18.46±0.85	20.13±2.18	14.02±0.70	16.97±1.81	19.55±1.88	12.63±1.51	10.10±1.42	15.72±1.96	15.88±2.31	11.97±1.32	13.81±1.78	
**Aldehydes**													
Hexanal	106.67±8.22	114.84±6.13	53.42±3.18	103.75±0.95	89.13±6.14	139.56±8.09	14.97±0.85	26.27±3.78	40.50±2.62	8.15±0.79	15.07±4.91	24.93±3.02	green-grassy, slightly fruity
(E)-2-Hexenal	129.25±6.79	133.52±9.42	52.64±2.62	169.82±11.77	113.59±9.44	88.61±3.17	31.71±2.74	36.63±0.55	68.60±4.35	23.80±2.40	38.79±3.78	37.22±6.91	green-fruity, fresh, grassy
2-Hexenal	--	--	--	--	--	--	--	1.50±0.41	--	--	--	--	green-fruity, fresh, grassy
3-Methyl-hexanal	0.03±0.00	--	0.02±0.00	--	0.04±0.02	--	--	--	--	--	--	0.05±0.01	
Heptanal	0.58 ±0.07	--	--	--	--	--	0.04±0.02	0.02±0.00	0.08±0.02	--	--	0.04±0.00	soapy, stale, citrus, rancid
(Z)-2-Heptenal	0.11±0.06	--	--	--	--	--	--	--	--	--	--	--	almond, mushroom, soapy
Benzaldehyde	0.59±0.18	--	0.81±0.16	--	--	0.54±0.38	--	--	--	0.08±0.05	0.19±0.10	0.93±0.05	sweet, almond, burnt, caramel
Nonanal	0.89±0.11	1.17±0.36	0.94±0.13	1.27±0.35	1.13±0.02	0.90±0.21	0.27±0.09	0.57±0.35	0.97±0.28	0.85±0.28	0.47±0.09	0.89±0.34	pungent, green, grassy, fresh
Decanal	--	0.39±0.10	--	--	--	--	--	0.15±0.09	--	0.27±0.03	--	0.19±0.06	sour, acidic, fruity, green, soapy
**Total Aldehyde content**	238.12±10.95	250.00±11.54	107.83±4.40	274.84±11.99	203.89±11.06	229.61±8.81	46.99±3.00	65.14±4.22	110.15±5.14	33.15±3.00	54.43±5.23	63.26±7.33	
**Esters**													
Butanoic acid, methyl ester	0.15±0.33	--	0.03±0.02	--	0.18±0.10	--	0.23±0.06	0.09±0.02	--	0.11±0.02	0.17±0.11	0.28±0.03	

Butanoic acid, 2-methyl ester	--	--	--	--	--	--	0.07±0.03	--	--	0.13±0.05	--	--	
Acetic acid, butyl ester	0.48±0.15	--	--	0.32±0.11	--	--	--	--	--	--	--	--	
Propanoic acid, pentyl ester	--	--	--	--	--	0.07±0.04	--	--	--	--	--	0.04±0.00	
Pentanoic acid, pentyl ester	--	--	--	--	--	--	--	--	0.70±0.06	--	0.06±0.01	--	
3-Methyl-1-butanol, acetate	19.85±1.04	17.03±1.90	11.27±2.35	45.58±3.49	33.82±2.17	28.76±6.02	5.57±0.87	23.77±2.21	38.99±3.16	38.83±5.23	15.60±0.25	17.17±2.29	wine, fruit, grass
Acetic acid, 2-ethylhexyl ester	0.41±0.11	--	0.89±0.29	0.26±0.17	0.31±0.12	0.20±0.15	0.34±0.08	0.59±0.38	0.34±0.06	0.93±0.49	0.22±0.15	0.26±0.09	fruit, Pear
**Total Esters content**	20.89±1.06	17.03±1.90	12.19±2.37	46.16±3.51	34.31±2.19	29.03±6.03	6.21±0.88	24.45±2.23	39.03±3.16	39.90±5.24	16.05±0.29	17.47±2.29	
**Ketones**													
3-Methyl-2-butanone	0.02±0.01	--	--	--	--	--	--	--	--	--	0.20±0.13	--	
4-Hydroxy-3-methyl-2-butanone	1.04±0.15	1.47±0.54	--	--	0.87±0.09	--	--	--	2.01±1.42	--	--	--	pungent
2-Pentanone	0.09±0.03	--	--	--	--	--	0.09±0.06	0.08±0.01	0.10 ±0.10	0.21±0.11	--	--	
3-Methyl-4-heptanone	0.04±0.01	--	--	0.08±0.00	--	0.06±0.02	0.03±0.00	--	--	--	0.01±0.00	--	
2-Octanone	1.32±0.36	--	--	--	--	0.57±0.11	0.49±0.38	0.55±0.16	0.53±0.25	0.45±0.18	0.35±0.12	1.48±0.80	mushroom, pungent, fruity, earthy
1-Octen-3-one	0.04±0.00	--	--	--	--	0.03±002	--	--	--	--	--	--	
m-Ethylacetophenone	0.39±0.12	1.97±0.61	0.37±0.17	1.32±0.49	1.34±0.28	1.59±0.99	0.66±0.13	0.91±0.32	2.12±0.11	--	--	--	
Total content	2.94±0.42	3.44±0.82	0.37±0.17	1.40±0.49	2.22±0.30	2.28±1.00	1.27±0.41	1.54±0.36	4.76±1.45	0.66±0.21	0.56±0.19	1.48±0.80	
**Alkenes**													

5-Methyl-1-heptene	--	--	--	--	--	0.43±0.17	--	--	0.07±0.02	--	--	--	
6-Methyl-1-heptene	--	--	--	--	--	--	0.10±0.03	--	--	--	--	--	
2-Methyl-3-nonene	--	--	--	--	0.26±0.07	0.14±0.21	0.09±0.04	--	--	--	--	--	
5-Methyl-4-nonene	0.03±0.00	--	0.24±0.06	0.64±0.14	0.24±0.18	--	0.27±0.12	0.48±0.028	0.45±0.29	0.38±0.01	0.10±0.04	0.26±0.18	
1-Tetradecene	3.35±0.33	--	8.17±0.82	3.44±0.37	7.64±2.23	5.93±1.16	2.73±1.14	1.43±0.26	--	24.03±6.05	3.86±0.23	2.02±0.28	
(E)-5-Tetradecene	--	--	0.16±0.05	--	0.49±0.18	0.20±0.01	0.18±0.05	0.42±0.37	0.38±0.04	1.69±0.52	0.15±0.04	--	
1-Pentadecene	1.41±0.85	5.76 ±0.03	4.71±0.99	1.30±0.53	--	2.99±0.23	--	5.33±0.08	--	1.01±0.30	--	--	
Hexadecane	--	--	--	--	--	--	0.29±0.16	--	1.01±0.03	2.85 ±0.49	--	--	
**Total Alkenes content**	4.79±0.91	5.76±0.03	13.27±1.28	5.38±0.63	8.62±2.25	9.78±1.19	3.66±1.18	7.66±0.46	1.91±0.30	29.96±6.18	4.11±0.24	2.28±0.18	
**Acids**													
Acetyloxy-acetic acid	--	--	0.75±0.19	--	--	--	--	--	0.35±0.03	1.43±0.57	--	--	apple
4-Oxo-Pentanoic acid	--	--	--	--	0.34±0.08	--	--	--	0.58±0.28	--	--	--	
n-Hexadecanoic acid	--	--	--	--	--	--	--	0.05±0.00	0.36±0.08	--	--	--	unpleasant smell
Total content	0.00±0.00	0.00±0.00	0.75±0.19	0.00±0.00	0.34±0.08	0.00±0.00	0.00±0.00	0.05±0.00	1.29±0.29	1.43±0.57	0.00±0.00	0.00±0.00	
**Others**													
2-Ethyl-furan	--	--	--	--	--	0.05±0.00	--	--	--	--	--	--	
Tetrahydro-2,5-dimethyl-furan	--	--	--	0.08±0.03	--	--	--	--	--	--	--	--	caramelized
Total content	0.00±0.00	0.00±0.00	0.00±0.00	0.08±0.03	0.00±0.00	0.05±0.00	0.00±0.00	0.00±0.00	0.00±0.00	0.00±0.00	0.00±0.00	0.00 ±0.00	

Note: --, not detected.

Table S4. Eigenvalues, variance contribution rate, cumulative contribution rate and component loading moment of principal components.

Quality indicators	Load coefficient	
	PC1	PC2
Sweetness score	0.960	0.214
Sourness score	0.783	0.169
Taste and bitter score	0.882	0.244
Overall satisfaction score	0.880	0.309
Firmness	-0.246	-0.369
SSC	0.758	-0.618
Soluble sugar	0.872	-0.096
TA	0.898	-0.265
Citric acid	0.603	-0.698
Malic acid	0.720	-0.582
Succinic acid	0.177	-0.896
Sugar-acid ratio	-0.763	0.260
Glucose	0.818	-0.514
Fructose	0.923	-0.078
TPC	0.308	0.838
TFC	0.203	0.912
DPPH	0.209	0.920
ABTS	0.097	0.653
TAC	0.063	0.884
Catechin	0.269	0.253
Chlorogenic acid	0.321	0.476

Quinic acid	0.554	-0.783
Rutin	0.178	0.896
Neochlorogenic acid	0.462	0.397
Epicatechin	0.487	0.506
(E)-2-Hexen-1-ol	-0.028	-0.025
2-Ethyl-1-hexanol	-0.232	-0.274
Hexanal	-0.501	-0.353
(E)-2-Hexenal	-0.416	-0.223
Nonanal	-0.368	-0.172
3-Methyl-1-butanol, acetate	-0.548	0.377
m-Ethylacetophenone	-0.711	-0.351
1-Pentadecene	0.062	-0.538
1-Tetradecene	0.255	0.469
Acetic acid, 2-ethylhexyl ester	0.328	0.008
6-Methyl-1-heptanol	0.172	-0.553
Eigenvalues	11.09	9.86
Variance contribution rate (%)	30.80	27.39
Cumulative contribution rate (%)	30.80	58.19
